# Saturation editing of *RNU4-2* reveals distinct dominant and recessive disorders

**DOI:** 10.1038/s41586-026-10334-9

**Published:** 2026-04-08

**Authors:** Joachim De Jonghe, Hyung Chul Kim, Ayanfeoluwa Adedeji, Elsa Leitão, Ruebena Dawes, Christina M. Kajba, Benjamin Cogné, Yuyang Chen, Alexander J. M. Blakes, Cas Simons, Rocio Rius, Javeria R. Alvi, Florence Amblard, Christina Austin-Tse, Sarah Baer, Elsa V. Balton, Pierre Blanc, Daniel G. Calame, Charles Coutton, Chloe A. Cunningham, Nitsuh Dargie, Katrina M. Dipple, Haowei Du, Salima El Chehadeh, Ian Glass, Joseph G. Gleeson, Olivier Grunewald, Paul Gueguen, Radu Harbuz, Marie-Line Jacquemont, Richard J. Leventer, Pierre Marijon, Olfa Messaoud, Tipu Sultan, Christel Thauvin, Catherine Vincent-Delorme, Elif Yilmaz Gulec, Julien Thevenon, Rodrigo Mendez, Daniel G. MacArthur, Christel Depienne, Caroline Nava, Nicola Whiffin, Gregory M. Findlay

**Affiliations:** 1 The Genome Function Laboratory, The Francis Crick Institute, London, UK; 2 Big Data Institute, University of Oxford, Oxford, UK; 3 Centre for Human Genetics, University of Oxford, Oxford, UK; 4 Department of Biochemical Engineering, University College London, London, UK; 5 Institute of Human Genetics, University Hospital Essen, University Duisburg-Essen, Essen, Germany; 6 Nantes Université, CHU de Nantes, CNRS, INSERM, L’Institut du Thorax, Nantes, France; 7 Nantes Université, CHU de Nantes, CNRS, INSERM, Génétique médicale, Nantes, France; 8 Manchester Centre for Genomic Medicine, Division of Evolution and Genomic Sciences, School of Biological Sciences, Faculty of Biology, Medicine and Health, University of Manchester, Manchester, UK; 9 Centre for Population Genomics, Garvan Institute of Medical Research, Sydney, New South Wales Australia; 10 Centre for Population Genomics, Murdoch Children’s Research Institute, Melbourne, Victoria Australia; 11 Department of Pediatric Neurology, University of Child Health Sciences, The Children’s Hospital, Lahore, Pakistan; 12 Service de Génétique, Génomique et Procréation, CHU Grenoble Alpes, Grenoble, France; 13 GCS AURAGEN, Lyon, France; 14 Université Grenoble Alpes, INSERM U 1209, CNRS UMR 5309, Institut for Advanced Biosciences, Grenoble, France; 15 Broad Center for Mendelian Genomics, Program in Medical and Population Genetics, Broad Institute of MIT and Harvard, Cambridge, MA USA; 16 Service de pédiatrie, Hôpitaux Universitaires de Strasbourg, Strasbourg, France; 17 Department of Medicine, University of Washington School of Medicine, Seattle, WA USA; 18 Laboratoire SeqOIA, Paris, France; 19 Section of Pediatric Neurology, Department of Pediatrics, Baylor College of Medicine, Houston, TX USA; 20 Texas Children’s Hospital, Houston, TX USA; 21 Victorian Clinical Genetics Services, Murdoch Children’s Research Institute, Melbourne, Victoria Australia; 22 Department of Paediatrics, University of Melbourne, Melbourne, Victoria Australia; 23 Department of Pediatrics, University of Washington, Seattle, WA USA; 24 Brotman Baty Institute for Precision Medicine, Seattle, WA USA; 25 Department of Molecular and Human Genetics, Baylor College of Medicine, Houston, TX USA; 26 Service de Génétique Médicale, Institut de Génétique Médicale D’Alsace, Hôpitaux Universitaires de Strasbourg, Strasbourg, France; 27 Laboratoire de Génétique Médicale, Institut de Génétique Médicale d’Alsace, INSERM UMRS_1112, CRBS, Université de Strasbourg, Strasbourg, France; 28 Rady Children’s Institute for Genomic Medicine, San Diego, CA USA; 29 Department of Neurosciences and Pediatrics, University of California, San Diego, San Diego, CA USA; 30 U1172-LilNCog-Lille Neuroscience and Cognition, CHU de Lille, Lille, France; 31 Laboratoire de Genopathies, CHU Lille, Lille, France; 32 Service de Génétique, CHRU de Tours, Tours, France; 33 Université de Tours, Imaging Brain and Neuropsychiatry iBraiN, Tours, France; 34 Centre de Référence Maladies Rares ‘Anomalies du Développement et Syndromes Malformatifs’, FHU Genomeds, CHRU de Tours, Tours, France; 35 Royal Children’s Hospital, Melbourne, Victoria Australia; 36 Harvard Medical School, Boston, MA USA; 37 Centre de référence maladies rares, Déficiences Intellectuelles de Causes Rares, Centre de Génétique, FHU-TRANSLAD, CHU Dijon Bourgogne, Dijon, France; 38 Unité Fonctionnelle Innovation en Diagnostic Génomique des Maladies Rares, Fédération Hospitalo-Universitaire-TRANSLAD, CHU Dijon Bourgogne, Dijon, France; 39 UMR1231 GAD, Inserm, Université Bourgogne-Franche Comté, Dijon, France; 40 Clinique de Génétique, Hôpital Jeanne de Flandre, CHU de Lille, Lille, France; 41 Consultation de génétique, CH Arras, Arras, France; 42 Department of Medical Genetics, Istanbul Medeniyet University Medical School, Istanbul, Turkey; 43 Medical Genetics Clinic, Istanbul Goztepe Prof Dr Suleyman Yalcin City Hospital, Istanbul, Turkey; 44 Cardiovascular Medicine, Stanford University, Stanford, CA USA; 45 Sorbonne Université, Institut du Cerveau—Paris Brain Institute—ICM, Inserm, CNRS, APHP, Département de Génétique, Hôpital de la Pitié Salpêtrière, Paris, France

**Keywords:** Neurodevelopmental disorders, Disease genetics, RNA splicing, Small RNAs, Mutagenesis

## Abstract

Recently, de novo variants in an 18-nucleotide region in the centre of *RNU4-2* were shown to cause ReNU syndrome, a syndromic neurodevelopmental disorder that is predicted to affect tens of thousands of individuals worldwide^1,2^. *RNU4-2* is a non-protein-coding gene that is transcribed into the U4 small nuclear RNA component of the major spliceosome^3^. ReNU syndrome variants disrupt spliceosome function and alter 5′ splice site selection^1,4^. Here we performed saturation genome editing (SGE) of *RNU4-2* to identify the functional and clinical impact of variants across the entire gene. The resulting SGE function scores, derived from variants’ effects on cell fitness, discriminate ReNU syndrome variants from those observed in the population and markedly outperform in silico variant effect prediction. Using these data, we redefine the ReNU syndrome critical region at single-nucleotide resolution, resolve variant pathogenicity for variants of uncertain significance and show that SGE function scores delineate variants by phenotypic severity and the extent of observed splicing disruption. Furthermore, we identify variants affecting function in regions of *RNU4-2* that are critical for interactions with other spliceosome components. We show that these variants cause a new recessive neurodevelopmental disorder that is distinct from ReNU syndrome. Together, this work defines the landscape of variant function across *RNU4-2*, providing critical insights for both diagnosis and therapeutic development.

## Abstract (web summary)

Saturation genome editing of *RNU4-2* identifies the functional and clinical impact of variants across the entire gene and delineates variants that cause a new recessive neurodevelopmental disorder distinct from ReNU syndrome.

## Main

The spliceosome is a large ribonucleoprotein complex that mediates RNA splicing. De novo variants in a gene encoding one of the small nuclear RNA (snRNA) components of the spliceosome, *RNU4-2*, were recently shown to cause ReNU syndrome, a prevalent neurodevelopmental disorder (NDD)^1,2^. ReNU syndrome is a complex multi-system disorder characterized by moderate to severe global developmental delay, intellectual disability, hypotonia, acquired microcephaly, speech and motor difficulties, low bone density and often seizures^1,4^.

*RNU4-2* encodes the U4 snRNA, which is a critical component of the major spliceosome. In particular, U4 is tightly bound with the U6 snRNA in the U4/U6.U5 tri-small-nuclear ribonucleoprotein and the U4/U6 duplex needs to be unwound for activation of splicing^3^. Variants identified in individuals with ReNU syndrome cluster in an 18-nucleotide (nt) region in the centre of *RNU4-2* that is depleted of variants in population datasets (the ‘critical region’, or CR)^1^. This region is known to accurately position U6 for recognition of the 5′ splice site. Consistent with this, variants causing ReNU syndrome have been shown to alter 5′ splice site usage^1^, with this disruption correlating with phenotype severity^4^. Similarly, variants in two distinct structures within the 18-nt CR (the T-loop and Stem III) have been proposed to differ in clinical severity^4^.

The precise relationship between genetic variation in *RNU4-2* and clinical impact remains incompletely characterized. The variants initially characterized in individuals with ReNU syndrome are all within the 18-nt CR; however, more recent work has proposed a role for variants outside this region, in the 5′ stem loop^5^. It is unclear which, if any, variants outside the CR could also cause NDD. This is particularly important as the increased mutation rate of *RNU4-2* and other snRNA genes means that there will be many chance occurrences of variants among sequenced individuals with syndromic NDD^6^. Up to 75% of individuals with ReNU syndrome have the same single-nucleotide insertion (n.64_65insT). Whether the high recurrence of this particular variant is due to ascertainment bias, germline selection and/or an increased mutation rate is at present unknown. Furthermore, it is unclear whether available variant effect predictors (for example, CADD^7^) can effectively distinguish between pathogenic and benign variants in *RNU4-2*.

Resolving these questions will be critical to ensure accurate, comprehensive diagnoses of individuals affected by ReNU syndrome. One approach to clarifying variant impact is through the generation of functional data of variant effect, which can mechanistically inform why specific variants cause disease and improve clinical interpretation of rare variants^8^. However, no experimental assay has yet been established to evaluate variants in *RNU4-2*, owing to its recent association with NDD.

Saturation genome editing (SGE) is a powerful approach to delineate genotype–phenotype relationships^9^. Crucially, it does not rely on variants being observed in an individual with or without disease. Instead, every possible variant across a gene or region can be engineered and the relative functional effects of each determined through a cellular readout. SGE experiments have been performed across numerous protein-coding genes, including *BRCA1*^10^, *CARD11*^11^, *DDX3X*^12^, *VHL*^13^ and *BAP1*^14^. In each case, the SGE assay has accurately differentiated between known pathogenic and benign variants.

Here, we perform SGE of the human *RNU4-2* noncoding RNA. We implemented an approach to combat the high sequence homology between *RNU4-2* and its many homologues and pseudogenes, obtaining a variant effect map that effectively distinguishes variants known to cause ReNU syndrome from those in population controls. We redefine the CR at single-nucleotide resolution, resolve pathogenicity assignments for variants of uncertain significance, and show that function scores for variants within the CR correlate closely with phenotypic severity. Furthermore, we identify functionally critical variants in other regions of *RNU4-2* that underlie a recessive NDD marked by clinical features that are distinct from those of ReNU syndrome.

## SGE maps the effects of *RNU4-2* variants

**Fig. 1:**
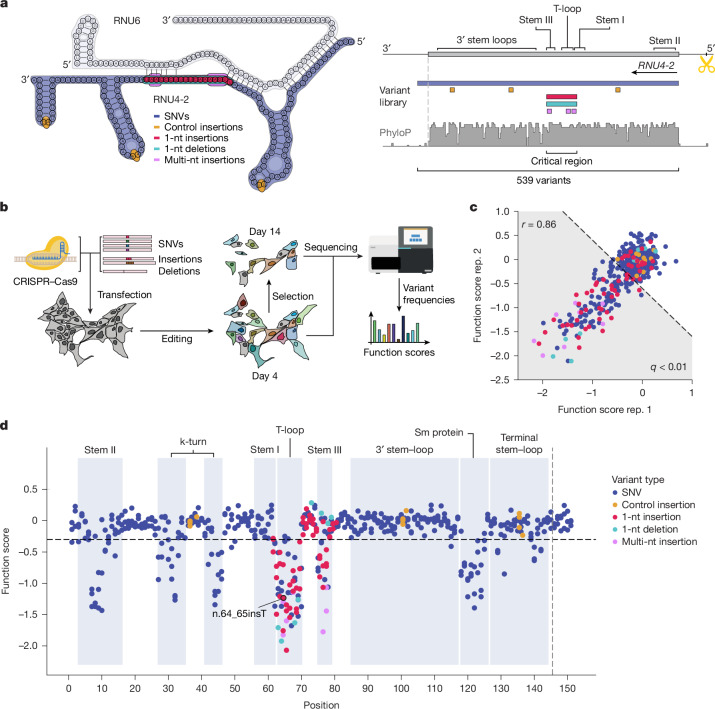
SGE reveals the functional spectrum of *RNU4-2* variants. **a**, Schematic of SGE library design and CRISPR targeting strategy for *RNU4-2*. Positions of library variants including all possible SNVs (navy; across the 145-nt transcript and 6-nt 3′), control 1-nt insertions in loop regions (yellow), CR 1-nt insertions (red) and deletions (teal) and multi-nt insertions (light purple) are denoted on a schematic of RNU4-2 and RNU6 in complex (left) and by genomic location (right). A gRNA was designed to cleave upstream of *RNU4-2* (scissors), avoiding highly repetitive sequence and allowing for a PAM-blocking variant to be installed in a region of low conservation (PhyloP 100 vertebrates basewise conservation track shown). **b**, Schematic of SGE experiments in HAP1. Following editing, cells were collected on days 4 and 14. Sequencing was performed to quantify variant frequencies at each timepoint and function scores were calculated. **c**, Function scores for 539 variants were correlated across biological replicates (Pearson’s *r* = 0.86 for replicates 1 and 2). The function score threshold delineating significantly depleted variants is indicated with the dashed line. **d**, Function scores are plotted by genomic position in relation to *RNU4-2* (RefSeq NR_003137.3). The line at n.145 marks the end of the transcript, with 18 more distal SNVs also scored. Points in **c**,**d** are coloured by variant type with a single legend included for these two panels. CRISPR–Cas9 icon in **b** adapted from Bioicons (https://bioicons.com/?query=CRISPR; CRISPR_Cas9 schematic), Marcel Tisch, under a Creative Commons licence CC0 1.0 Universal.

Performing SGE on regions of high sequence homology poses a challenge in that the protocol requires CRISPR–Cas9 editing of a single locus, specific amplification of the edited locus from millions of cells and accurate variant calling from amplicon sequencing. Alignment of *RNU4-2* (RefSeq NR_003137.3) to *RNU4-1* (RefSeq NR_003925.1) reveals mismatches at only 4 of the 145 nt. The sequence upstream of *RNU4-2*, however, is both unique and poorly conserved across species, such that guide RNAs (gRNAs) predicted to be highly specific^15^ can be designed in conjunction with protospacer adjacent motif (PAM)-disrupting edits to block Cas9 recutting (Fig. 1a).

Lacking established models for assaying *RNU4-2* variants, we chose to perform SGE in HAP1 cells, a haploid human line in which growth effects have accurately distinguished pathogenic variants across several protein-coding genes^10,12–14,16,17^. To HAP1 cells lacking LIG4 (HAP1-LIG4-KO), we codelivered Cas9 with a gRNA directing DNA cleavage 31-nt upstream of *RNU4-2* to install a library comprising 539 variants by homology-directed DNA repair (HDR). The library included all possible single base substitutions from the first transcribed nucleotide to 6 nt beyond the most 3′ position of the RNU4-2 transcript (GRCh38, chr12:120291753–120291903), as well as all 1-nt deletions and insertions in the CR, including all but one variant known to cause NDD (omitting n.72_73del, which was reported after assay design; Fig. 1a). Uncertain whether pathogenic variants would show phenotypes in the HAP1-based assay, we included 8 2-nt to 5-nt insertions at positions in the CR previously associated with disease, reasoning these may have strong effects. As negative controls, we included 12 1-nt insertions in stem loops outside the CR, which were not predicted to be deleterious (Supplementary Table 1).

**Extended Data Fig. 1:**
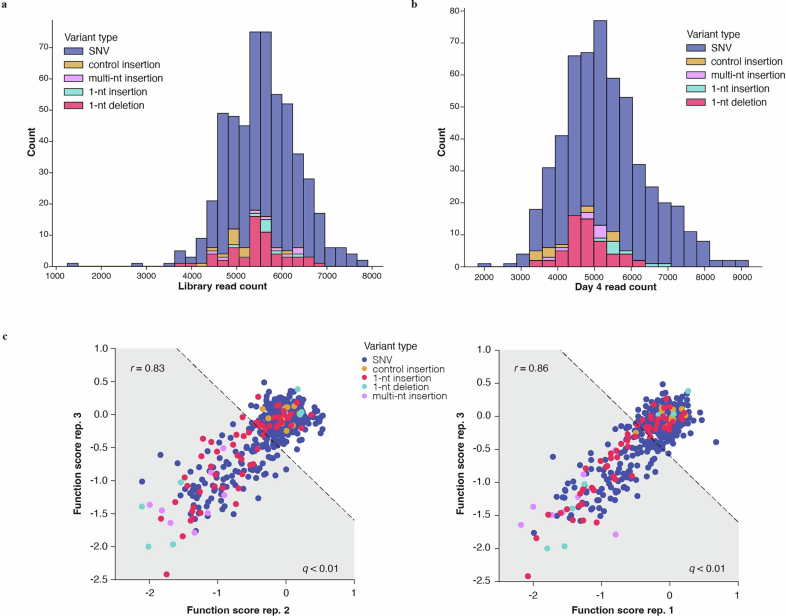
Quality control metrics for *RNU4-2* SGE experiments. **a**, The distribution of variant read counts in the HDR library is plotted for all *n* = 539 variants included in library design. Of reads from the HDR library, 0.0068% and 4.6% matched unedited reference and PAM-edit only, respectively. **b**, The distribution of variant read counts in day 4 gDNA is plotted, with counts averaged across biological replicates. **c**, Inter-replicate function score correlations are plotted, with Pearson’s *r* shown and variants coloured by mutation type.

Adapting an optimized SGE protocol for HAP1 cells^13^ (Fig. 1b), we successfully scored all variants included in the library, observing an average of 52% editing by HDR at day 4. Editing was confirmed by sequencing to be specifically targeted to *RNU4-2*, and not *RNU4-1*. Function scores, reflecting variants’ effects on growth (Methods), were highly correlated across three biological replicates (Pearson’s *r* = 0.83–0.86; Fig. 1c and Extended Data Fig. 1). As expected, given their location in the U4/U6 secondary structure, all 12 negative control variants scored near 0 (mean, −0.009, s.d. = 0.11). We defined a neutral distribution from these negative controls to identify 151 significantly depleted variants (*q* < 0.01, that is, function score less than −0.302). The 8 multi-nucleotide insertions in the CR included as positive controls all were depleted, with function scores ranging from −0.73 to −1.82. Mapping variants’ function scores to their linear transcript position reveals that depleted variants are clustered, rather than distributed evenly across the gene (Fig. 1d).

## SGE data resolve variant pathogenicity

**Fig. 2:**
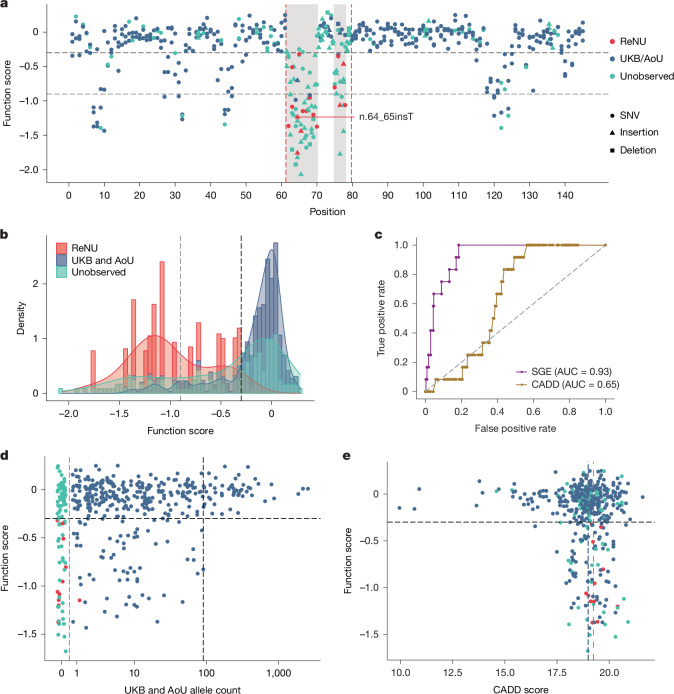
Function scores accurately discriminate variants underlying ReNU syndrome. **a**, Function scores for 521 variants within the RNU4-2 transcript are plotted by position and coloured by their association with ReNU syndrome (red), presence in the UK Biobank (UKB) or All of Us (AoU) cohorts (blue) or absence from both cohorts (teal). Depleted variants within the 18-nt CR (vertical red dashed lines) are confined to two smaller regions (shaded grey) and include all ReNU syndrome variants scored (*n* = 18). These regions, n.62-70 and n.75-78, correspond closely to the T-loop and Stem III regions, respectively. The black dashed line (function score −0.302) indicates significantly depleted variants and the grey dashed line (function score −0.90) separates ‘moderate’ from ‘strong’ depletion. **b**, Stacked histogram and overlaid density plot of function scores by category comparing 18 ReNU syndrome variants with 353 variants in UK Biobank and/or All of Us and 150 unobserved variants. **c**, ROC curves show the performance of function scores and CADD scores for classifying 12 ReNU syndrome SNVs from 346 SNVs observed at least once in population controls. **d**, Function scores for SNVs are plotted by combined UK Biobank and All of Us allele count. Higher allele counts were correlated with higher function scores (Spearman’s *ρ* = 0.29, two-sided *P* = 2.8 × 10^−11^). Among the 50 most frequently observed SNVs (combined allele count greater than 91; black dashed line), no SNVs were depleted. The grey dashed line separates absent variants (combined allele count of 0) from those observed at least once (combined allele count greater than 0). **e**, Function scores for the 435 tested SNVs are plotted by CADD score. The dashed line at *y* = −0.302 indicates significantly depleted SNVs, whereas the red line at *x *= 19.25 and the blue line at *x* = 18.99 indicate median CADD scores for ReNU syndrome SNVs and SNVs present in population cohorts, respectively.

We annotated all assayed variants within *RNU4-2* with whether or not they had been observed in individuals with ReNU syndrome^1^, observed in population cohorts (UK Biobank^18^ or All of Us), or observed in neither (unobserved; Fig. 2a). All 18 variants observed in ReNU syndrome were depleted in the assay (function score less than −0.302), whereas 81.0% (286 out of 353) of population variants scored as normal (function score −0.302 or more; Fig. 2b). Accordingly, function scores effectively discriminate between ReNU syndrome variants and those identified in the population (Fig. 2c; area under the receiver operating characteristic (ROC) curve (AUC) of 0.93). Most variants that are unobserved in population cohorts score normally (56.0%; 84 out of 150); however, many are as, or even more, depleted than ReNU syndrome variants. Specifically, the four variants with the lowest function scores are all unobserved (Supplementary Table 1).

**Extended Data Fig. 2:**
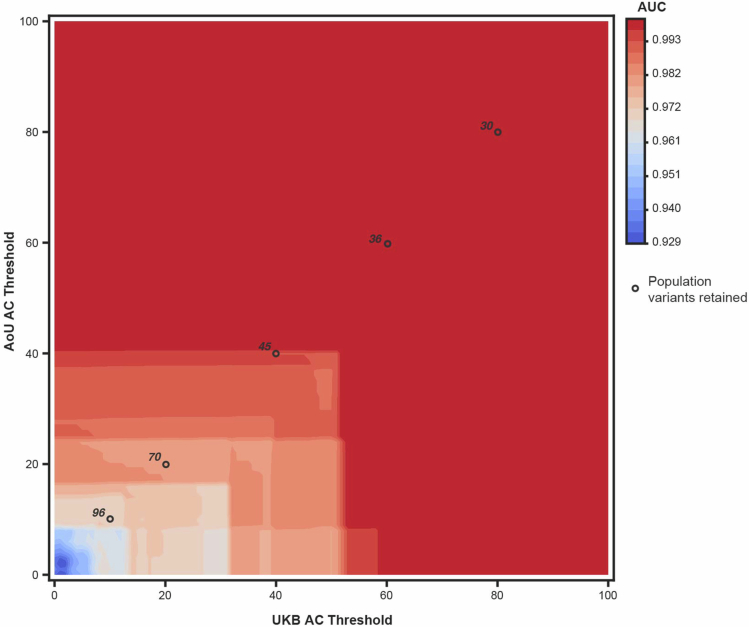
ReNU syndrome variants are discriminated with high precision from variants seen frequently in population controls. ROC-AUC measurements for distinguishing 12 ReNU syndrome SNVs from population control SNVs by SGE score are displayed as a heatmap. Each AUC was determined using only variants in UK Biobank and All of Us with allele counts above the thresholds indicated on the axes. For select allele count thresholds applied to both cohorts (10, 20, 40, 60, and 80), the number of population variants retained for the ROC-AUC calculation is indicated.

We observed a significant correlation between single-nucleotide variant (SNV) allele counts in population cohorts and function scores, with rarer SNVs tending to be more depleted by SGE (Spearman’s *ρ *= 0.29, *P* = 2.8 × 10^−11^; Fig. 2d). Among the 50 SNVs with the highest combined allele counts in the UK Biobank and All of Us cohorts, none were depleted in the assay. Indeed, applying more stringent allele count thresholds to define control variants in population cohorts consistently improved the assay’s classification performance (Extended Data Fig. 2). These findings indicate that depleted variants observed in population cohorts are unlikely to be the result of experimental noise and, instead, represent genuine variants affecting *RNU4-2* function segregating in the general population.

**Extended Data Fig. 3:**
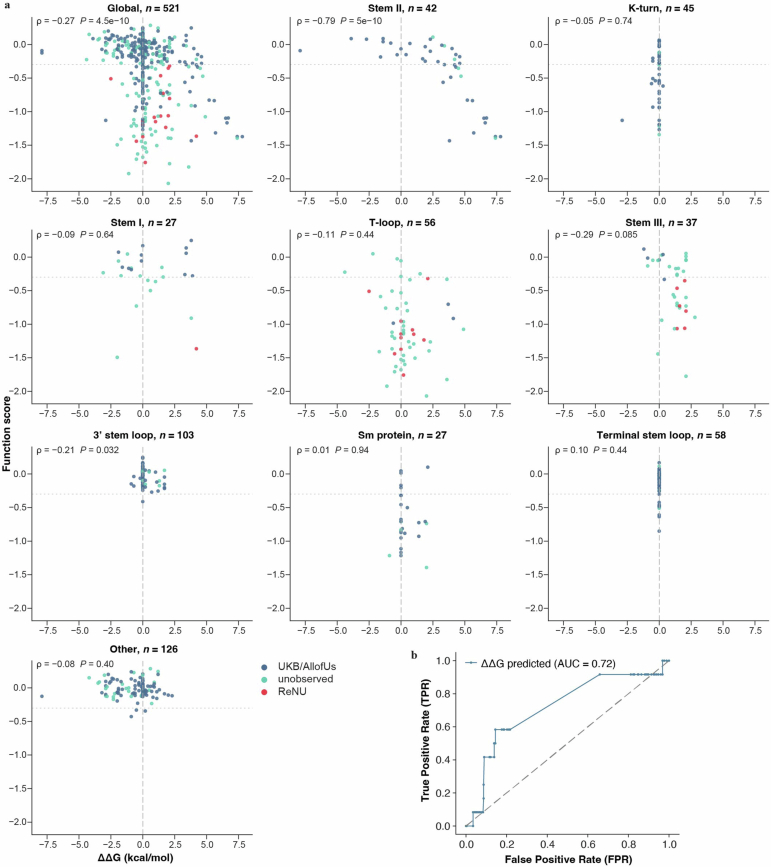
Correlations between function scores and predicted effects on RNA binding stability. ViennaRNA was used to predict the effects of variants (*n* = 521) on the minimum free energy of U4/U6 RNA binding compared to reference (ΔΔG). **a**, Predicted ΔΔG values are plotted versus function scores for the whole transcript, as well as for individual regions (Spearman’s ρ). **b**, ROC curve for classifying ReNU syndrome variants from population controls using ViennaRNA-predicted ΔΔG values (AUC = 0.72).

The discriminatory power of our SGE assay was substantially greater than that of the genome-wide in silico tool CADD^19^ (Fig. 2c; AUC = 0.65). Given the high conservation of the entire *RNU4-2* gene, most SNVs have very similar CADD scores (Fig. 2e). Although CADD scores for ReNU syndrome SNVs are marginally higher on average than those for SNVs in population cohorts (ReNU median 19.2; UK Biobank and All of Us median 19.0; one-sided Wilcoxon *P* = 0.040), a CADD score threshold that would capture all ReNU syndrome SNVs (18.89 or greater) would also annotate 56.4% (195 out of 346) of SNVs observed in UK Biobank and All of Us, and 55.6% (183 out of 329) of SNVs with normal SGE function scores, as probably deleterious. By contrast, our SGE function score threshold of −0.302 captures all ReNU syndrome SNVs and only 19.1% (66 out of 346) of SNVs observed in population cohorts. We also observe only a weak correlation of SGE function scores with changes to U4/U6 RNA binding stability predicted by ViennaRNA (*ρ* = −0.27, *P* = 4.5 × 10^−10^; Extended Data Fig. 3a). The observed effect is limited to specific regions, most notably Stem II (*ρ *= −0.79, *P* = 5.0 × 10^−10^). By contrast, no significant correlation is observed in the T-loop or Stem III and, overall, ΔΔ*G* values from ViennaRNA do not classify ReNU syndrome variants as well as SGE (ROC-AUC 0.72 versus 0.93, respectively; Extended Data Fig. 3b).

**Extended Data Fig. 4:**
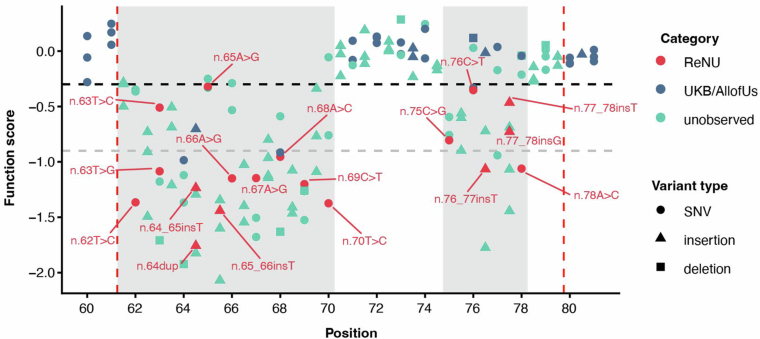
Function scores for variants within the *RNU4-2* critical region. Function scores are plotted by position and coloured by their association with ReNU syndrome (red), presence in the UK Biobank or All of Us cohorts (blue), or no observation in either (teal). Variants score lowly in two regions within the CR (shaded), n.62-70 and n.75-78, which correspond to the T-loop and Stem III, respectively. The black dashed line (function score = −0.302) indicates significantly depleted variants and the gray dashed line (function score = −0.90) separates “moderate” from “strong” depletion. The vertical red dashed lines represent the boundaries of the 18 nucleotide ReNU CR reported by Chen et al.^1^ drawn to include insertions at n.61_62 and n.79_80.

The assay clearly delineates the 18-nt CR of *RNU4-2* (Fig. 2a) within which variants cause ReNU syndrome; however, some variants in this region score normally. Using these data, we redefine the CR to two smaller regions of 9 nt (n.62-70, inclusive of insertions at n.61_62) and 4 nt (n.75-78), corresponding to the T-loop and Stem III, respectively (Extended Data Fig. 4). Although the T-loop region matches that reported by ref. ^2^, the CR overlapping Stem III is 3-nt smaller than previously suggested. Within these two regions, 85.4% (76 out of 89) of tested variants (79.5% of SNVs), including all ReNU syndrome variants, have significant function scores, compared with 17.4% (75 out of 432) across the remainder of *RNU4-2*.

**Extended Data Fig. 5:**
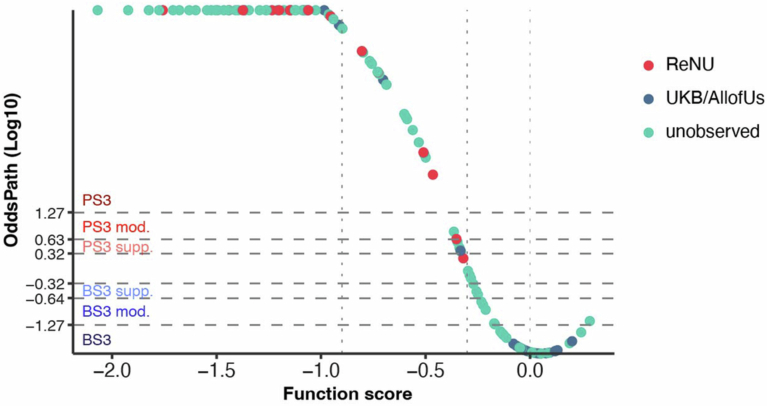
Calibration of function scores to evidence for clinical classification of variants in relation to ReNU syndrome. Gaussian mixture modelling was used to estimate odds of pathogenicity (OddsPath). Function scores are plotted against OddsPath values for *n* = 127 variants within the ReNU syndrome critical region. Vertical dotted lines mark the median of insertion controls (x = 0), as well as thresholds for “moderate” (−0.302) and “strong” (−0.90) depletion. Horizontal dashed lines indicate OddsPath thresholds for assigning evidence strengths in accordance with ACMG guidelines^8^. OddsPath values are capped for variants with function scores below −1.0 to display all points.

We next used our function scores to assign evidence strengths for clinical variant classification^8^. We deemed the 17 pathogenic or likely pathogenic variants reported in ref. ^4^ and assayed here to be associated with ReNU syndrome and 45 variants with combined allele counts across the UK Biobank and All of Us above 100 to be neutral. A Gaussian mixture model was then applied to determine the odds of pathogenicity (OddsPath) for each variant (Methods, Extended Data Fig. 5 and Supplementary Table 1). Within the CR, 69 of 127 (54.3%) variants receive PS3 strong evidence of pathogenicity, including 16 of 18 variants reported to be pathogenic, with the other two variants receiving PS3 moderate or indeterminate evidence. A further 38 (29.9%) variants receive BS3 strong evidence of benignity. As no variants outside the CR have been associated with ReNU syndrome, we refrain from assigning evidence strengths to variants outside the CR.

Recent work by one research group^4^ classified three variants outside the CR and one deletion within the CR as variants of uncertain significance. Three of these variants were included in our assay (n.76del, n.92C>G and n.111C>T) and all three had normal function scores (0.12, 0.04 and 0.05, respectively). Notably, all three variants are also observed in population controls. Furthermore, a recent paper proposed a link between two 5′ stem loop variants, each identified in a single individual and inherited from an unaffected mother, and ReNU syndrome^5^. One of these variants is included in our assay (n.30A>T), and its score of −0.305 just crosses the threshold to be classified as depleted; however, other depleted variants in the same region are observed in population controls. Finally, of two variants recently associated with retinitis pigmentosa^20^, the one that is included in our assay (n.56T>C) has a normal function score (−0.23).

## SGE depletion predicts disease severity

**Fig. 3:**
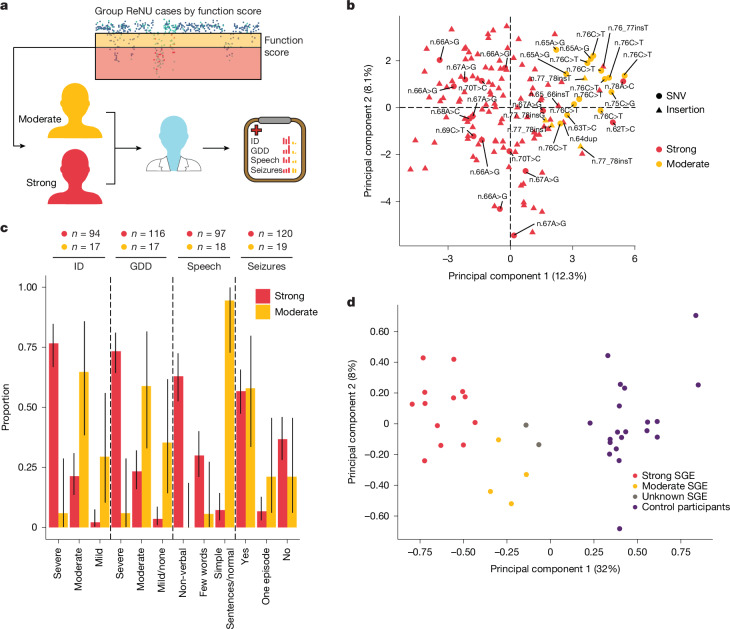
Function scores predict ReNU syndrome severity and degree of splicing disruption. **a**, Schematic showing how ReNU variants are split into two categories based on their SGE function score: strong depletion (function score less than −0.9; red) and moderate depletion (−0.9 < function score < −0.302; yellow). **b**, The first two principal components from clustering of 143 ReNU syndrome cases by phenotype using the approach from ref. ^4^. Individuals are coloured by their variant SGE function score class. Unlabelled triangles indicate occurrences of n.64_65insT. **c**, The proportion of affected individuals with each phenotype is plotted, with cases grouped by SGE function score class. The number of individuals (*n*) in each comparison group is shown for each phenotype. Error bars indicate 95% confidence intervals centred on each proportion (capped at 0 and 1.0). Full data, including statistics for comparisons between groups, are included in Extended Data Table 1. **d**, Principal component analysis based on PSI values from significant 5′ splice site events detected from RNA sequencing data using rMATS, comparing 19 patients with ReNU with 20 control participants (purple), as performed in ref. ^4^. Individuals with ReNU are coloured by their variant SGE function score class. GDD, global developmental delay; ID, intellectual disability.

**Extended Data Fig. 6:**
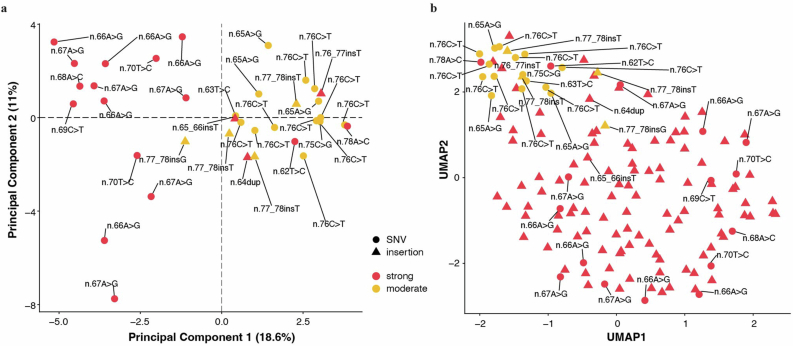
Phenotype clustering of ReNU patients. **a**, PCA clustering as in Fig. 3a but removing individuals with the recurrent n.64_65insT variant. **b**, Phenotype clustering of all individuals represented in Fig. 3a using a UMAP representation.

A previous study proposed a difference in phenotypic severity between ReNU syndrome variants mapping to the T-loop and Stem III structures of the U4/U6 duplex^4^. This difference is seen in our data, with Stem III variants having on average, higher function scores (T-loop mean −1.13; Stem III mean −0.75; one-sided Wilcoxon *P* = 0.012). However, we also observe considerable variation in function scores for ReNU variants within each of the two regions. For example, two SNVs within the T-loop, n.63T>C and n.65A>G, have function scores above the mean observed for Stem III variants (−0.51 and −0.32, respectively). To investigate this, we repeated the phenotype clustering analysis of 143 individuals with ReNU syndrome from ref. ^4^. We classified the variants into two categories corresponding to ‘moderate’ (−0.9 < function score < −0.302) and ‘strong’ (function score less than −0.9) levels of depletion in the assay (Fig. 3a and Extended Data Fig. 4). All of the individuals with moderate category variants cluster together, including the four individuals with the n.63T>C (*n* = 1) and n.65A>G (*n* = 3) T-loop variants (Fig. 3b). These results remained consistent when excluding n.64_65insT from the analysis (that is, the result is not driven by the recurrent insertion variant alone) and when using a uniform manifold approximation and projection (UMAP) representation (Extended Data Fig. 6).

To further determine whether SGE function scores were able to discriminate between more severe and milder ReNU syndrome variants, we compared four specific phenotypes. Individuals with variants in the strong depletion group were significantly more likely to have severe developmental delay (73.3% versus 5.9%; odds ratio = 42.7; 95% confidence interval (CI) 6.1–1,841.8; two-sided Fisher’s *P* = 1.1 × 10^−7^), severe intellectual disability (76.6% versus 5.9%; odds ratio = 50.4; 95%CI 7.1–2,197.0; two-sided Fisher’s *P* = 3.6 × 10^−8^) and absent speech or to speak only a few words (92.8% versus 5.6%; odds ratio = 195.5; 95%CI 24.7–8,591.7; two-sided Fisher’s *P* = 6.6 × 10^−14^) than individuals with moderate depletion variants. There was no difference in the occurrence of seizures between variant groups (Fig. 3c and Extended Data Table 1).

To test whether the strength of SGE depletion also correlates with the extent of splicing disruption observed in individuals with ReNU syndrome, we repeated a second analysis from ref. ^4^. We regenerated a principal component analysis of percentage spliced-in (PSI) values for 5′ splice sites that differed significantly in usage between ReNU cases and control participants. Individuals with strong and moderate SGE function scores clustered separately, with the strong variant individuals being more distant from control participants (Fig. 3d).

## A recessive NDD linked to *RNU4-2* variants

Seventy-five variants outside the ReNU CR are depleted in the SGE assay (Supplementary Table 1). Unlike the depleted variants in the ReNU CR, most of these other depleted variants (84.0%; 63 out of 75) are observed in population control cohorts, albeit at low frequencies (Fig. 2a). To investigate whether these variants are associated with NDD-related traits, we compared individuals heterozygous for such variants (*n* = 592) and individuals with non-depleted SNVs (*n* = 12,374) in *RNU4-2* with individuals without any variants in *RNU4-2*, using the UK Biobank. We did not find any significant differences in fluid intelligence scores, childhood developmental disorder diagnoses or age of leaving education (Extended Data Table 2).

Because our SGE assay was performed in a haploid cell line, we reasoned that depleted variants outside the CR may instead be associated with recessive phenotypes. We searched global rare disease cohorts and identified 20 individuals, with biallelic depleted variants: 10 (including 3 pairs of siblings) with homozygous variants and 10 (including 4 pairs of siblings) who were each concordant for compound heterozygous depleted variants (Extended Data Table 3). None of these variants were located in the ReNU CR, yet all 20 individuals had NDD phenotypes. None of the individuals had an existing genetic diagnosis that fully explained their observed phenotypes (Methods). Across the rare disease cohorts, no individuals with phenotypes unrelated to NDD had biallelic depleted variants. Only a single individual across the UK Biobank and All of Us cohorts is homozygous for a SGE-depleted variant (n.31T>G, function score −0.730). This individual has only primary level education (highest grade, one to four) and reports difficulties with ‘dressing or bathing’, ‘doing errands alone’ and ‘concentrating, remembering or making decisions’, consistent with a possible intellectual disability.

The clinical phenotypes of the 20 identified NDD individuals are characterized as part of a broader cohort (total *n* = 38) in a companion paper^21^. The 18 extra individuals reported in this broader cohort all have biallelic *RNU4-2* variants, but at least 1 variant had a non-significant function score or was not scored with SGE. In brief, we define a new NDD characterized by global developmental delay, intellectual disability, delayed or absent speech, hypotonia, spasticity, microcephaly, ophthalmological and visual impairments and seizures, with variable involvement of genitalia, skin, hair and limb anomalies. On MRI, individuals show distinctive white matter abnormalities and cerebellar atrophy that are not seen in ReNU syndrome^21^.

**Fig. 4:**
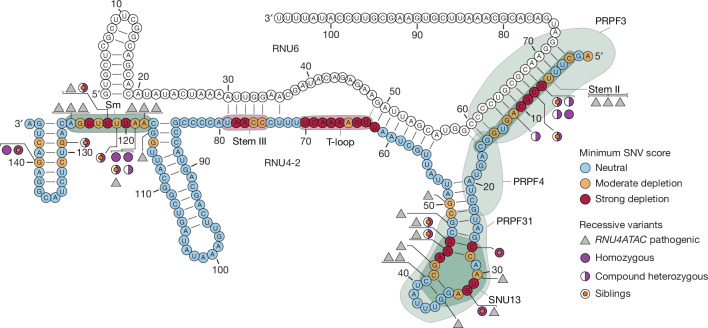
SGE-depleted variants outside the CR cause a recessive NDD. The lowest SGE function score class among SNVs at each position is indicated on the U4/U6 secondary structure. Outside the CR, low SGE scores occur at positions of spliceosomal protein binding, indicated by teal shaded regions. Grey triangles correspond to homologous positions of *RNU4ATAC* at which (likely) pathogenic variants have been linked to recessive disease (from ClinVar; Supplementary Table 2). *RNU4-2* variants with low function scores observed in recessive NDD cases are indicated, with filled purple circles indicating variants observed as homozygous and half-filled circles indicating variants observed in the compound heterozygous state. An orange dot in the centre of a circle indicates that the variant is observed in two affected siblings. Six (likely) pathogenic *RNU4ATAC* variants could not be confidently assigned to an equivalent nucleotide in *RNU4-2*. Three of these (n.8C>A, n.13C>T and n.16G>A) are shown together as mapping to Stem II. The other three (n.29T>G, n.30G>A and n.111G>A) are not shown.

Depleted variants outside the ReNU CR broadly map to four regions of U4/U6 secondary structure that are known to mediate interactions between U4 and other components of the spliceosome: (1) the central portion of the Stem II interaction with U6 from nucleotides 6 to 11 (ref. ^3^); (2) a ‘k-turn’ structure required for protein binding^22,23^ comprising nucleotides 27 to 33 and nucleotides 42 to 46; (3) a region from nucleotides 118 to 126 that interacts with a ring of Sm proteins that are important for U4 biogenesis and stability^24,25^ and (4) a portion of the terminal stem loop formed by base-pairing of nucleotides 129 to 131 with nucleotides 140 to 142 (Fig. 4). All variants identified in the 20 recessive NDD cases map to these four regions. Variants in structurally equivalent regions of *RNU4ATAC*, which encodes the minor spliceosome equivalent of U4, U4atac, cause rare recessive RNU4atac-opathies^26–28^. Of the 13 unique *RNU4-2* variants identified in the recessive NDD cases, 5 have exact equivalents in *RNU4ATAC* that are (likely) pathogenic in ClinVar (n.32G>A, n.45G>C, n.46G>A, n.119A>G and n.122T>G; Supplementary Table 2). They include n.119A>G (function score −0.686; *RNU4ATAC* equivalent n.117A>G; ClinVar variation ID 1525441), which was homozygous in two individuals and compound heterozygous in three individuals, including two brothers.

**Extended Data Fig. 7:**
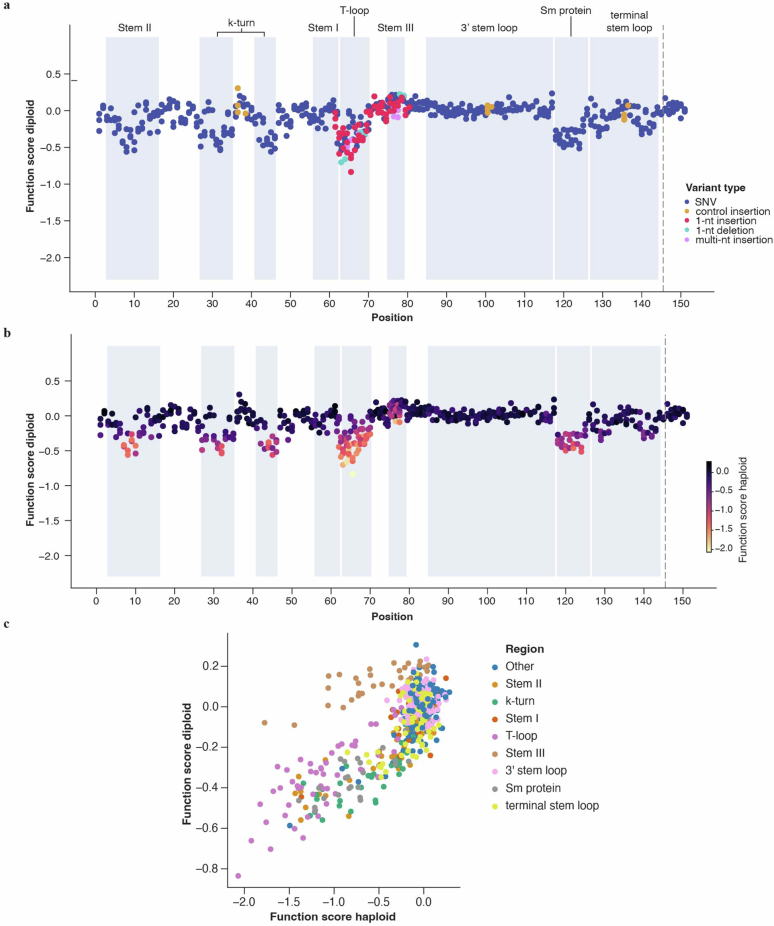
Correlation of the SGE assay in haploid versus diploid HAP1 cells. **a**, Function scores (*n* = 539) from SGE in diploid HAP1 cells, plotted by transcript position and coloured by variant type. **b**, Function scores from SGE in diploid HAP1 cells coloured by the function score from SGE in haploid HAP1 cells. **c**, Correlation of function scores in diploid versus haploid HAP1 cells, coloured by the region in which each variant is located (Pearson’s *r* = 0.75).

In an attempt to distinguish recessive and dominant variants experimentally, we performed SGE of *RNU4-2* once more, this time using a diploid population of HAP1 cells selected through fluorescence-activated cell sorting (Methods). This experiment revealed function scores to be attenuated across the gene due to the presence of the second allele (Extended Data Fig. 7a,b and Supplementary Table 1). However, all variants assayed in the Stem III region scored neutrally in diploid HAP1, suggesting pathogenic Stem III variants probably affect cell fitness in a manner that is distinct from pathogenic variants elsewhere. For all other regions, function scores between haploid and diploid models were highly correlated (Extended Data Fig. 7c), indicating fitness effects in diploid HAP1 cells do not delineate dominant and recessive variants in vivo.

## Discussion

*RNU4-2* was the first noncoding RNA to be identified as having a substantial contribution to the prevalence of NDD, with ReNU syndrome predicted to affect around 100,000 individuals worldwide^1,2^. Here we developed an SGE assay to systematically assess the function of variants across *RNU4-2* and map genotype–phenotype relationships. We show that function scores accurately identify variants underlying ReNU syndrome and can distinguish these variants by disease severity. Furthermore, we define the CR at the centre of *RNU4-2* within which variants cause dominant ReNU syndrome, at nucleotide resolution. In two regions, of 9 nt and 4 nt, 85.4% of all tested variants are depleted. However, some variants in these regions, particularly in Stem III, have normal function scores and are therefore unlikely to be pathogenic. As a consequence, these data have immediate use in clinical interpretation of newly observed variants in individuals with NDD. Indeed, calibration of the SGE function scores for use within the ACMG/AMP framework in the context of ReNU syndrome showed that these data can be used to give strong evidence towards either a pathogenic or benign classification.

We identified four regions of the U4/U6 duplex structure, outside the ReNU CR where variants are also depleted. This led us to uncover a new recessive NDD caused by homozygous and compound heterozygous variants in these regions that were depleted in SGE. This NDD is described comprehensively in ref. ^21^, in which we also expand the cohort to include 38 individuals with biallelic *RNU4-2* variants: the 20 individuals presented here with significant function scores for both variants, and 18 extra individuals harbouring variants in the same functional regions with at least one variant that was not significantly depleted or not assayed by SGE. Through comprehensive clinical phenotyping and analysis of RNA sequencing data, we show that the recessive NDD is phenotypically and mechanistically distinct from ReNU syndrome. For example, MRI findings in individuals with ReNU syndrome most commonly include enlarged ventricles and corpus callosum abnormalities^4^, whereas individuals with biallelic *RNU4-2* variants commonly have progressive white matter changes and cerebellar atrophy. Although we cannot yet determine the prevalence of the recessive NDD, SGE-depleted variants outside the ReNU CR are found in 0.12% and 0.094% of individuals in the UK Biobank and All of Us cohorts, respectively. Hence, the recessive NDD is rarer than ReNU syndrome, but the prevalence is likely increased in populations with higher rates of consanguinity^21^.

Distinct mechanisms underlie dominant and recessive *RNU4-2*-associated NDDs. We previously showed that individuals with ReNU syndrome have an increase in use of alternative non-canonical 5′ splice sites^1^, consistent with the role of the T-loop and Stem III regions in accurately positioning the U6 ACAGAGA sequence to receive the 5′ splice site. Recessive *RNU4-2* variants map to different locations within U4, outside the T-loop and Stem III. They are found in key regions of binding between U4 and other important spliceosome factors. The same regions have previously been shown to be important in U4 mutational analyses in yeast^25^ and variants in the 5′ stem loop k-turn that we identify as depleted occur at nucleotides that are essential for SNU13/15.5k protein binding in vitro^23^. In our companion paper^21^, we show through analysis of blood RNA sequencing data that individuals with biallelic *RNU4-2* variants do not have the ReNU signature of disrupted 5′ splice site selection. Furthermore, biallelic individuals have notably decreased *RNU4-2* expression, which is not observed in individuals with ReNU syndrome, supporting a distinct loss-of-function molecular mechanism. As variants in the equivalent regions and nucleotides of *RNU4ATAC* that cause recessive RNU4atac-opathies have been shown to lead to intron retention^29,30^, a similar mechanism may underlie recessive *RNU4-2* NDD. However, this was not readily evident in RNA sequencing analysis in blood^21^.

*RNU4-2* is a striking example of genetic pleiotropy, with variants in different regions of the RNA, which is only 145 nt in length, causing both two distinct NDDs and retinitis pigmentosa. This adds complexity to variant interpretation and makes it particularly important to calibrate functional evidence with consideration of underlying mechanisms. Although we showed that function scores for variants within the ReNU CR can provide strong evidence for clinical interpretation, we were unable to calibrate our assay for variants outside the ReNU CR due to a lack of independently defined pathogenic variants in these regions^8^, as all individuals with recessive NDD were identified on the basis of function score. Whereas we anticipate that our SGE data will prove highly useful for delineating variant pathogenicity for recessive disease, until orthogonal calibration can be performed, we recommend PS3 supporting evidence be assigned to significantly depleted variants outside the CR. It is important to note that we set a relatively conservative threshold to define significantly depleted variants (*q* < 0.01) using synthetic controls in the absence of bona fide benign variants. Although all variants associated with ReNU syndrome scored below this threshold, we cannot exclude the possibility that variants with more subtle effects may be clinically relevant, particularly in relation to recessive disease. We cannot fully exclude the possibility that variants that score just below the −0.302 function score threshold are benign and represent false positives. The calibration of function scores to evidence strength for ReNU variant classification reflects this, as variants were not assigned PS3 strong evidence in favour of pathogenicity unless their function scores were below −0.45.

Thus far, there are no strong data linking variants outside the CR to dominantly inherited NDD. This is supported by our analysis of heterozygous SGE-depleted variants outside the CR in the UK Biobank, in which we do not find any associations with intellectual disability related phenotypes. Accordingly, SGE data should not be used as evidence for the pathogenicity of variants for dominantly inherited ReNU syndrome beyond the CR. We note that the 5′ stem loop variants n.30A>T (function score −0.305) and n.43_44insT have been putatively associated with NDD^5^, with a link initially proposed with dominant ReNU syndrome. However, these variants are within the ‘k-turn’ region linked to recessive disease in this study, and both are inherited from unaffected parents. Furthermore, n.43_44insT is identified in an individual with NDD in our companion paper, as compound heterozygous with a variant in Stem II^21^. Collectively, these data indicate that 5′ stem loop variants are more likely to lead to recessive NDD than dominant ReNU syndrome.

Our HAP1-based SGE assay has several limitations. Most notably, the growth-based readout does not inform directly on underlying mechanisms of splice alteration (for example, altered 5′ splice site usage, intron retention). This means that in the haploid context, both dominant and recessive effects are observed, which cannot be separated by function score alone. We also performed SGE in diploid HAP1 cells. Whereas function scores from these experiments revealed differences between T-loop and Stem III variants, they were once more unable to distinguish dominant and recessive variants in vivo. It is likely that specific changes in splicing underlying certain clinical phenotypes may not occur in HAP1 due to differences between cell types. It is notable, for instance, that a variant recently associated with retinitis pigmentosa (n.56T>C) did not score significantly. Furthermore, most individuals with ReNU syndrome (70–75%) have the same single base insertion, n.64_65insT. Our data indicate that this variant is not unique in its functional severity, with many variants scoring similarly or having even lower function scores. This result could argue against high recurrence being the result of a particularly damaging functional effect driving ascertainment, suggesting that positive selection in the female germline or an increased local mutation rate might be more likely explanations. However, we cannot rule out the possibility that this variant leads to unique changes in splicing not reflected in SGE function scores.

Future experiments using more cell types will be valuable for delineating mechanisms of *RNU4-2* pleiotropy. Likewise, testing larger insertions and deletions both inside and outside the ReNU CR will add insights into the degree of tolerated disruption across different regions of *RNU4-2*. For example, in ref. ^4^, the authors identified a 2-nt deletion (n.72_73del) in 2 individuals. This variant falls between Stem III and the T-loop but suggests that larger insertions and deletions in this region may also be disruptive to these structures. As we have observed for CR variants associated with ReNU syndrome, the degree of functional impact caused by recessive NDD variants may correlate with disease severity. There may also be phenotypic differences between individuals with variants mapping to the four distinct regions we identified. Thorough phenotyping of large cohorts of cases will be necessary to establish how the degree of functional effect influences phenotype.

In summary, this work illustrates the power of a variant effect map for a locus recently implicated in disease to discover new genotype–phenotype associations and understand mechanisms underlying disease. SGE data for *RNU4-2* will be critical for accurately diagnosing patients with at present unexplained NDD and provide insights that are valuable for efforts to design effective therapies. Finally, the SGE strategy we used to overcome the high sequence homology of *RNU4-2* can be replicated to dissect other snRNAs recently linked to disease^31,32^.

## Methods

### Single guide RNA design and cloning

The gRNA used for SGE was designed using Benchling’s CRISPR design tool to search the *RNU4-2* locus, including upstream and downstream regions of low sequence homology to *RNU4-1* and pseudogenes, identifying a candidate with high on-target and low off-target scores. The selected gRNA was not predicted to target *RNU4-1*, owing to eight mismatches occurring in the protospacer and PAM. The gRNA spacer sequence was ligated into the pX459 backbone as previously described^33^. In brief, complementary primers containing the spacer were ordered from IDT (Supplementary Table 3), phosphorylated, hybridized and ligated into the pX459 linearized backbone followed by PlasmidSafe DNase (Lucigen) digestion. Next, 2 µl of the ligation reaction were transformed in NEB Stable Competent *Escherichia coli* cells using the high-efficiency transformation protocol and 75 µl of transformant was plated on ampicillin-resistant plates and cultured overnight at 30 °C. Three colonies were then picked and grown overnight at 37 °C in 7 ml of Luria–Bertani medium supplemented with carbenicillin (100 µg ml^−1^). Plasmid DNA was extracted using the QIAprep Spin Miniprep kit (Qiagen) and verified using Plasmidsaurus whole-plasmid sequencing. The selected clone was then grown in 100 ml of Luria–Bertani medium at 37 °C in a shaking incubator supplemented with carbenicillin. The cells were then pelleted and the plasmid was extracted using a ZymoPure Maxiprep kit (Zymo Research), endotoxins were removed using EndoZero columns (Zymo Research) and the product was quantified with the Qubit double-stranded DNA (dsDNA) BR assay kit (Invitrogen).

### HDR library cloning

An oligonucleotide library comprising *RNU4-2* variants was manufactured by Twist Bioscience and subsequently cloned into a vector containing homology arms for *RNU4-2* to make the HDR library for SGE.

To generate the vector with homology arms, a nested PCR was performed on genomic DNA (gDNA) extracted from HAP1 cells^10^ using primers designed to generate homology arms of 700–800 base pairs (bp) flanking *RNU4*-2 (Supplementary Table 3). The PCR was performed using the Kapa HiFi HotStart ReadyMix (Roche). The product was purified using AmpureXP (Beckman Coulter) magnetic beads at 1.2× volume and eluted in 12 µl of nuclease-free water. The amplicon containing *RNU4-2* homology arms was then inserted in the linearized pUC19 backbone using In-Fusion HD cloning (Takara) and 2 µl of cloning reaction was transformed into NEB Stable cells following the manufacturer’s 5-min transformation protocol. Cells were plated on agar plates containing ampicillin and incubated at 30 °C overnight. The pUC19 plasmid containing *RNU4-2* homology arms (pUC19-RNU4-2-HA) was purified and sequence-verified from a successfully transformed clone. pUC19-RNU4-2-HA was then diluted to 8.7 pg in a 50-µl PCR reaction and amplified with Kapa HiFi to obtain a linearized product with 17–18 bp complementarity to the *RNU4-2* oligo library. A PAM-blocking mutation was introduced 27 nt upstream of the *RNU4-2* sequence (chromosome 12:120291930-C-G) by means of primer overhang extension during PCR. The location of the PAM-disrupting edit was selected to minimize recutting by Cas9, converting a 5′-GGG PAM sequence to 5′-GCG. The PAM-disrupting edit had a CADD score of 4.20 (Phred) and a 100 vertebrates PhyloP score of 0.11. The reaction was treated with 1 µl of DpnI (NEB) for 30 min at 37 °C, gel extracted and quantified. Then, the *RNU4-2* oligo library was amplified using Kapa HiFi and purified using AmpureXP (1.2×). The amplified library and linearized pUC19-RNU4-2-HA plasmid were then assembled using the In-Fusion HD cloning kit, and the product was transformed into NEB Stable cells using the high-efficiency transformation protocol. To quantify efficiency, 1% of cells in the transformation reaction were plated and the remainder were cultured in 100 ml of Luria–Bertani medium with carbenicillin overnight at 37 °C. Cells were then pelleted by centrifugation and the final *RNU4-2* HDR library was extracted using the ZymoPure Maxiprep kit (Zymo Research) with endotoxin removal. The isolated HDR library was quantified with a Qubit dsDNA BR assay kit and sequence-verified by Plasmidsaurus.

### HAP1 cell culture

HAP1 cells used for SGE (the HAP1-LIG4-KO line; herein referred to as ‘HAP1’) show increased rates of editing by HDR due to a frameshifting mutation in *LIG4* (ref. ^10^). Frozen HAP1 cells were thawed at 37 °C in a water bath, then supplemented with 10 ml of prewarmed Iscove’s Modified Dulbecco’s Medium (IMDM) containing l-glutamine, 25 nM HEPES (Gibco), 10% FBS (Gibco), 1% penicillin–streptomycin (Gibco) and 2.5 μM 10-deacetyl-baccatin-III (DAB, Stratech), herein referenced to as IMDMc. Cells were centrifuged at 300*g* for 3 min. The supernatant was then aspirated and the cells were resuspended in fresh media, plated on a 10-cm dish and cultured at 37 °C with 5% CO_2_. The next day, the IMDMc media was replaced, and cells were cultured routinely from that point forward.

The HAP1 subculture routine included a 1:5 split every 48 h or 1:10 split every 72 h to prevent cells from exceeding 80% confluency. To split cells, the media was aspirated and the dish washed with 10 ml of room-temperature Dulbecco’s PBS (Gibco). Following Dulbecco’s PBS aspiration, the cells were treated with 1 ml of 0.25% trypsin–EDTA (Gibco) and incubated for 3 min at 37 °C. Next 14 ml of prewarmed IMDMc was then added and cells were collected and centrifuged at 300*g* for 5 min. Cells were then resuspended in 10 ml of IMDMc, counted and seeded on a 10-cm dish.

### Generation of diploid HAP1 cells

Parental HAP1 cells were cultured for 9 days after thawing in IMDMc without DAB supplementation to allow for the spontaneous occurrence of diploid cells. On day 10, cells were stained with 5 µg ml^−1^ Hoechst working solution (Thermo Fisher Scientific) for 1 h at 37 °C, followed by fluorescence-activated cell sorting to select diploid cells using a BD FACSAria Fusion Flow Cytometer. Diploid cells were sorted on the basis of their G2/M peak (4n), with gates established using a monoclonal diploid HAP1 control population. Sorted diploid HAP1 cells were then expanded for 10 days in IMDMc without DAB supplementation before the subsequent SGE experiment.

### Transfection and selection

The day before transfection, 12 million cells were seeded on a 10-cm dish for each replicate and 2 million cells were seeded on a six-well plate for the negative control sample. On the day of transfection (day 0), a transfection mix containing 10 µg of HDR library, 30 µg of the pX459 gRNA plasmid and 24 µl of Xfect polymer (Takara) in a final volume of 800 µl was prepared according to the manufacturer’s instructions for each replicate. For the negative control sample, a pX459 plasmid with a gRNA targeting *HPRT1* (ref. ^13^) instead of *RNU4-2* was used to prevent successful editing, and the transfection volume mix was scaled down eightfold. Following transfection, cells were incubated for 24 h at 37 °C and supplemented with prewarmed IMDMc with 1 µg ml^−1^ puromycin (Cayman Chemical). On day 4, half of the cells for each replicate were collected for gDNA extraction and stored as a pellet at −70 °C; the rest were kept in culture in 15-cm dishes supplemented with 15 ml of IMDMc. The negative control sample was collected when reaching 70% confluency at day 6. A second sample of 10 million cells per replicate was collected at day 14 and stored at −70 °C.

### Sequencing library preparation

gDNA was extracted from cells using QIAshredder (Qiagen) columns followed by the Allprep DNA/RNA kit (Qiagen) according to the manufacturer’s instructions. Concentrations were determined using the Qubit dsDNA BR assay kit. The *RNU4-2* locus was subsequently amplified using nested PCR to avoid amplification of plasmid DNA, followed by an indexing PCR, in total using three primer sets (Supplementary Table 3). For the first reaction, the total gDNA template from each condition was partitioned into separate reactions, each containing 1.25 µg of DNA in a 100 µl reaction volume, using NEBNext Ultra II Q5 master mix (NEB) supplemented with MgCl_2_ (Ambion) to a final concentration 4 mM. The amplification reaction was monitored by quantitative PCR (qPCR) using SYBR green (Invitrogen) and stopped before completion. The reactions for each sample were pooled and mixed before 50 µl of each product was purified using AmpureXP (1.2×) and eluted in 15 µl of nuclease-free water. Then 1 µl of purified product was loaded into the second qPCR reaction (50 µl final volume) and amplified using NEBNext Ultra II Q5. The reaction was again monitored using SYBR green and stopped before completion. The AmpureXP purification was then repeated, and a final qPCR (NEBNext Ultra II Q5) to incorporate sample indexes and sequencing adapters was performed using 1 µl of purified product as template in a 50 µl reaction for 8 cycles. Final products were purified and quantified with the Qubit dsDNA HS kit. The samples were then pooled for sequencing, aiming for 5 million reads per experimental replicate timepoint, 2 million reads for the negative control sample and 1 million reads for the HDR library. The pool was purified using AmpureXP (1×), quantified and loaded on a Novaseq X sequencer (Illumina).

### Variant frequency quantification

The fastq files were de-multiplexed using the bcl2fastq script and the variants were quantified as previously described^13^. In brief, paired-end reads were adapter trimmed and merged, and reads containing N bases were discarded. HDR editing rates were computed from fastq files directly as the fraction of reads containing the exact PAM-blocking mutation. Fastq files were then aligned to a reference *RNU4-2* sequence and the frequency of each variant included in the library was determined.

### Function score calculation

All variants were observed in the library and day 4 at a frequency higher than 10^−4^, and were therefore included in downstream analyses. Function scores for library variants were first calculated per replicate, computed as the log_2_ ratio of day 14 to day 4 variant frequencies, normalized by subtracting the median function score of negative control insertions from all scores. Final function scores were then calculated for each variant by averaging function scores across replicates, again normalizing to the median of negative control insertions such that the median final function score of control insertions equals 0. For each variant, *P* values were determined using the norm.cdf function in Python, defining a normal distribution from the mean and standard deviation of function scores for negative control insertions. The *P* values were corrected for multiple hypothesis testing using the multipletests function in Python (Benjamini–Hochberg procedure) to derive *q* values. Significantly depleted variants were defined as those with *q* < 0.01, corresponding to a function score below −0.302. We further classified depleted variants into two categories using an arbitrary function score threshold of −0.9 to include sufficient variants and individuals per category to assess for phenotypic differences.

### Variant scoring with CADD and ViennaRNA

Variants were annotated as ReNU syndrome variants if they were reported in ref. ^1^ or classified as pathogenic or likely pathogenic in ref. ^4^. Variants were annotated with whether or not they were observed in the 490,640 genome sequenced individuals from the UK Biobank^18^ (DRAGEN pipeline) or in 414,840 individuals from All of Us V8. CADD v.1.7 (ref. ^19^) annotations were obtained by uploading a synthetic VCF to the online annotation tool (https://cadd.gs.washington.edu/score). As we preselected which insertions and deletions to include in the SGE assay (because of assay size limitations), we restricted analyses involving CADD to SNVs within the RNU4-2 transcript.

For variants assayed within the RNU4-2 transcript, predicted changes in U4/U6 interaction stability (ΔΔ*G*_bind_) were computed using the ViennaRNA package^34^ (v.2.7.0). Minimum free energies (MFEs) were obtained by use of RNA.fold_compound() at 37 °C using default Turner RNA thermodynamic parameters. U4/U6 pairing was modelled with the ViennaRNA cofold grammar by providing sequences in the dimer format (u4(AGCUUUGCGCAGUGGCAGUAUCGUAGCCAAUGAGGUUUAUCCGAGGCGCGAUUAUUGCUAAUUGAAAACUUUUCCCAAUACCCCGCCAUGACGACUUGAAAUAUAGUCGGCAUUGGCAAUUUUUGACAGUCUCUACGGAGACUGA).

+ ‘&’ + u6(GUGCUCGCUUCGGCAGCACAUAUACUAAAAUUGGAACGAUACAGAGAAGAUUAGCAUGGCCCCUGCGCAAGGAUGACACGCAAAUUCGUGAAGCGUUCCAUAUUUU), and the intermolecular MFE was extracted using mfe_dimer(). Single-strand MFEs for U4 and U6 were computed independently using mfe().

Binding free energy was defined as:

$$\Delta {G}_{{\rm{bind}}}=\Delta {G}_{{\rm{complex}}}-(\Delta {G}_{{\rm{U}}4}+\Delta {G}_{{\rm{U}}6})$$

The same procedure was applied to *RNU4-2* variant sequences, and differential stability was then calculated as:

$$\Delta \Delta {G}_{{\rm{b}}{\rm{i}}{\rm{n}}{\rm{d}}}=\Delta {G}_{{\rm{b}}{\rm{i}}{\rm{n}}{\rm{d}}.{\rm{v}}{\rm{a}}{\rm{r}}{\rm{i}}{\rm{a}}{\rm{n}}{\rm{t}}}-\Delta {G}_{{\rm{b}}{\rm{i}}{\rm{n}}{\rm{d}}.{\rm{r}}{\rm{e}}{\rm{f}}{\rm{e}}{\rm{r}}{\rm{e}}{\rm{n}}{\rm{c}}{\rm{e}}}$$

Positive ΔΔ*G*_bind_ values indicate predicted destablization of U4/U6 pairing.

Variants were mapped to the following structural regions of *RNU4-2*: Stem II (n.3 to n.16), k-turn within the 5′ Stem loop (n.27 to n.35 and n.41 to n.46), Stem I (n.56 to n.62), T-loop (n.63 to n.70), Stem III (n.75 to n.79), 3′ Stem loop (n.85 to n.117), Sm protein (n.118 to n.126) and terminal Stem loop (n.127 to n.144).

ROC area under the curve (AUC) values were calculated by assigning a 1 label to ReNU syndrome SNVs and a 0 label for SNVs observed in UK Biobank or All of Us. The labels and corresponding function scores were used to compute false positive and true positive rates (using Python’s roc_curve function), as well as ROC-AUC values (using the roc_auc_score function). This analysis was also restricted to SNVs only.

### Assigning evidence codes to variants based on function score

We followed established guidelines^8^ to calibrate function scores from SGE experiments in haploid cells to evidence strengths for classification of ReNU syndrome variants. To do so, we defined a gold standard set of pathogenic, dominantly inherited variants as the 17 previously reported^4^ as ‘pathogenic’ or ‘likely pathogenic’ for which we derived function scores. Few *RNU4-2* variants have been deemed benign in ClinVar, so we instead used reported allele counts in the UK Biobank and All of Us studies to define a neutral set of variants. This included all 45 assayed variants with a combined allele count of more than 100 between the two studies. A two-component Gaussian mixture model was then fit from the function score distributions of these variant sets, using the ‘Mclust’ package in R. This model was then used to determine the probability of pathogenicity for each variant in the CR based on function score. The resulting posterior probabilities were then converted to OddsPath values using a uniform prior of 0.5, and evidence codes were assigned according to established OddsPath thresholds^8^ with the exception that PS3 evidence was capped at strong (+4 points), in line with the limited number of gold standard variants available for calibration. We did not apply the model to variants outside the CR on account of there being no known pathogenic variants for ReNU syndrome in these regions.

### Phenotype severity and clustering

Categorical data for 44 clinical features from 143 patients with pathogenic and likely pathogenic *RNU4-2* variants^4^ were transformed into a 0–1 scale, with 0 indicating a more favourable phenotype and 1 a more severe presentation. Principal component analysis was generated after imputing missing data with 0 and performing variable scaling. UMAP representation was created using the umap package in R. Two-sided Fisher’s tests with Bonferroni adjustment to account for four tests were used to compare clinical features between SGE function score variant categories (strong versus moderate) in Extended Data Table 1.

### RNA sequencing cluster analysis

RNA sequencing from cultured lymphocytes was performed following the protocol described in ref. ^4^ for *RNU4-2* and rMATS-turbo (v.4.3.0)^35^ was run on 19 ReNU samples and 20 control participants (excluding one individual previously deemed a control in ref. ^4^ who was here found to be a recessive *RNU4-2* case); 101 significant alternative non-canonical 5′ splice sites (A5SS) events (false discovery rate less than 0.1, ΔPSI > 0.05) were retained. Then rMATS-turbo was rerun on the 19 ReNU samples, the 20 control participants, without statistical or ΔPSI filtering. The A5SS output was filtered on the 101 retained events and the PSI values were extracted to perform the principal component analysis.

### Association testing in UK Biobank

We extracted phenotypes associated with educational attainment from UK Biobank following an approach published previously^36^. Fluid intelligence scores (field ID 20016) were retrieved for all participants. Where many scores were recorded, the median value was taken. Age left education was calculated as the maximum value in age completed full time education (field ID 845). Diagnosis with childhood developmental disorder was defined using the ICD codes for intellectual disability (ICD-10: F70–F73, F78, F79; ICD-9: 317, 318, 319), epilepsy (ICD-10: G40), global developmental disorders (ICD-10: F80–F84, F88–F95, R62, R48, Z55; ICD-9: 299, 312, 313, 314, 315) and congenital malformations (ICD-10: Q0–Q99, ICD-9: 740–759).

We identified UK Biobank participants with: (1) depleted variants in the 18-bp *RNU4-2* CR (*n* = 6), (2) depleted variants outside the CR (*n* = 50) and (3) participants with non-depleted SNVs outside the CR (*n* = 12,132). We performed multiple linear regression on fluid intelligence scores and age left education, and multiple logistic regression on childhood developmental disorder for variant groups (2) and (3) defined above, compared with all individuals without any variants in any of the three groups. Age at recruitment (field ID 21022), age^2^ (age at recruitment × age at recruitment), sex (field ID 31) and first ten genetic principal components (field ID 22009) were included as covariates. *P* values were false discovery rate-corrected using the Benjamini–Hochberg method.

### Investigating *RNU4ATAC* variants in ClinVar

Variants in *RNU4ATAC* with classifications of pathogenic, likely pathogenic, pathogenic or likely pathogenic, benign, likely benign or benign or likely benign were downloaded from the ClinVar^37^ website on 4 March 2025. Two regions of *RNU4-2* and *RNU4ATAC* with identical structures were defined, mapping to the k-turn (*RNU4-2* nucleotides 26–52; *RNU4ATAC* nucleotides 31–57) and the Sm protein binding site (*RNU4-2* nucleotides 115–126; *RNU4ATAC* nucleotides 113–124). Variants at the same nucleotide in the structure and where the reference bases in *RNU4-2* and *RNU4ATAC* are identical, were marked as ‘equivalent’.

### Identifying biallelic variants in cohorts

We searched rare disease cohorts for individuals with biallelic variants in *RNU4-2*. These cohorts included the Genomics England 100,000 Genomes Project and NHS Genomic Medicine Service datasets accessed through the UK National Genomic Research Library^38^, the SeqOIA and Auragen clinical cohorts in France (PFMG2025), the Undiagnosed Disease Network, the Broad Institute Center for Mendelian Genomics and GREGoR (Genomics Research to Elucidate the Genetics of Rare Diseases)^39^ Consortium cohorts. We only included individuals with homozygous variants with function scores less than −0.302, or compound heterozygous variants in which both had function scores less than −0.302 (*n* = 20). All individuals had previous genome analysis including investigation of variants in known NDD genes and large structural variants. One individual (individual 17) had a reported likely pathogenic variant in *GLI3*; however, this variant did not explain all of their reported phenotypes (see ref. ^21^ for more details).

### Ethics

Informed consent was obtained for all participants included in this study from their parent(s) or legal guardian, with the study approved by the local regulatory authority. The 100,000 Genomes Project Protocol has ethical approval from the Health Research Authority Committee East of England Cambridge South (Research Ethics Committee ref. ^14^/EE/1112). This study was approved by Genomics England under Research Registry Projects 354. Health related research in UK Biobank was approved by the Research Ethics Committee under reference 20/NW/0274 with this research conducted under application number 81050.

We received an exception to the Data and Statistics Dissemination Policy from the All of Us Resource Access Board to report questionnaire response data for the single individual with a homozygous depleted variant as well as variant counts below 20 for all variants in *RNU4-2*.

### Reporting summary

Further information on research design is available in the Nature Portfolio Reporting Summary linked to this article.

## Online content

Any methods, additional references, Nature Portfolio reporting summaries, source data, extended data, supplementary information, acknowledgements, peer review information; details of author contributions and competing interests; and statements of data and code availability are available at 10.1038/s41586-026-10334-9.

## Supplementary information

Reporting Summary

Supplementary Table 1 Details and function scores for *RNU4-2* variants assayed with SGE.

Supplementary Table 2 List of variants in *RNU4ATAC* in ClinVar.

Supplementary Table 3 Oligonucleotide sequences used in this study.

Peer Review File

## Figures and Tables

**Extended Data Table 1:**
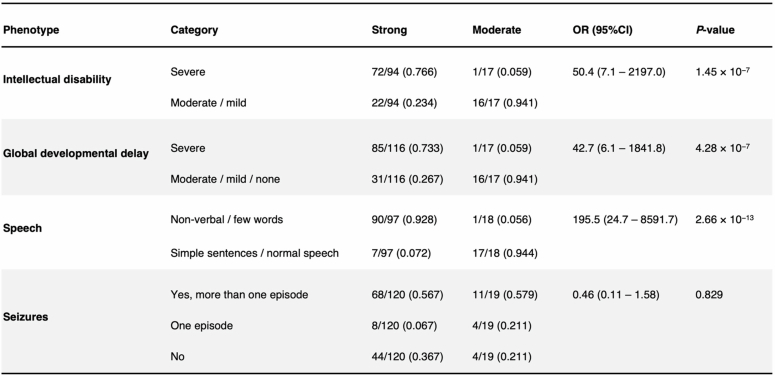
Comparison of clinical features by function score categories

Two-sided Fisher’s tests were used to compare intellectual disability (severe vs mild/moderate), developmental delay (severe vs moderate/mild/none), speech ability (non-verbal/few words vs simple sentences/normal speech), and epilepsy/seizures (yes/one episode vs no) between strong vs moderate depleted variants in the SGE assay. *P* values are Bonferroni adjusted for four tests.

**Extended Data Table 2:**
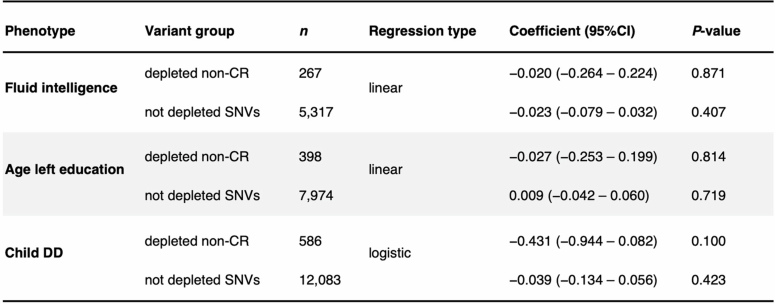
Results from association testing with intelligence-related metrics in the UK Biobank

Individuals with depleted variants outside of the ReNU syndrome critical region (non-CR; max *n* = 586), and individuals with SNVs with normal SGE function scores (≥ −0.302; max *n* = 12,083) were compared to individuals without variants in *RNU4-2* (fluid_intelligence *n* = 207,505; age left education *n* = 311,341, childhood developmental disorder (DD) = 472,252). The *n* shown in the table represents the number of individuals in each variant group without missing data that were included in each test.

**Extended Data Table 3:**
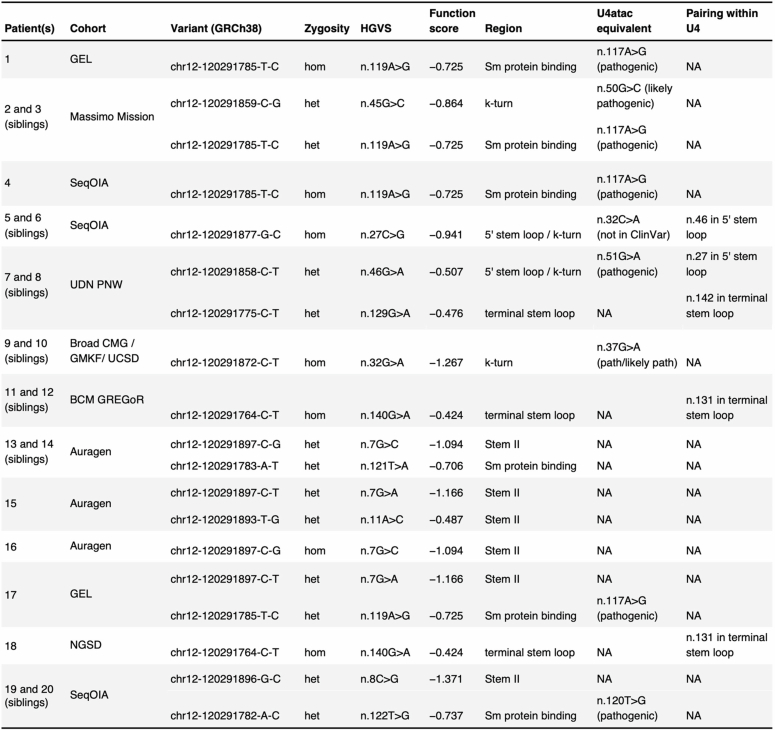
Homozygous and compound heterozygous variants in individuals with undiagnosed neurodevelopmental disorders

Equivalent variants in *RNU4ATAC* and their ClinVar classification are included for variants at the equivalent nucleotide and with the same reference (see Methods). Where a variant is part of a stem region of pairing within the U4 structure, the base it pairs with is noted.

## Data Availability

SGE data including all *RNU4-2* function scores are available in Supplementary Table 1. Fastq files from SGE experiments are available through the European Nucleotide Archive at accession PRJEB87505. RNA sequencing data (Fig. 3d) were taken from ref. ^4^ and are available in the European Genome–Phenome Archive at http://www.ebi.ac.uk/ega; study accession EGAS50000000889. UK Biobank and All of Us V8 data are available to researchers on approval of application (https://www.ukbiobank.ac.uk/use-our-data/apply-for-access/; https://www.researchallofus.org/).

## Code availability

Custom scripts used to analyse SGE experiments and generate figures are available at GitHub (https://github.com/FrancisCrickInstitute/RNU4-2_Saturation_Genome_Editing).
