# Supplementary material for: Saturation editing of RNU4-2 reveals distinct dominant and recessive disorders
Source: Nature. 2026 Apr 8;654(8118):429–36. doi: 10.1038/s41586-026-10334-9 (PMC13253345; doi:10.1038/s41586-026-10334-9)
Supplement: Supplementary file 1 — Reporting Summary [file 41586_2026_10334_MOESM1_ESM.pdf]

nature portfolio

Corresponding author(s): Nicola Whiffin, Gregory M. Findlay

Last updated by author(s): Feb 22, 2026

Reporting Summary

Nature Portfolio wishes to improve the reproducibility of the work that we publish. This form provides structure for consistency and transparency in reporting. For further information on Nature Portfolio policies, see our Editorial Policies and the Editorial Policy Checklist.

Statistics

For all statistical analyses, confirm that the following items are present in the figure legend, table legend, main text, or Methods section.

n/a	Confirmed	
☐	☒	The exact sample size (*n*) for each experimental group/condition, given as a discrete number and unit of measurement
☐	☒	A statement on whether measurements were taken from distinct samples or whether the same sample was measured repeatedly
☐	☒	The statistical test(s) used AND whether they are one- or two-sided<br>*Only common tests should be described solely by name; describe more complex techniques in the Methods section.*
☐	☒	A description of all covariates tested
☐	☒	A description of any assumptions or corrections, such as tests of normality and adjustment for multiple comparisons
☐	☒	A full description of the statistical parameters including central tendency (e.g. means) or other basic estimates (e.g. regression coefficient) AND variation (e.g. standard deviation) or associated estimates of uncertainty (e.g. confidence intervals)
☐	☒	For null hypothesis testing, the test statistic (e.g. *F*, *t*, *r*) with confidence intervals, effect sizes, degrees of freedom and *P* value noted<br>*Give P values as exact values whenever suitable.*
☒	☐	For Bayesian analysis, information on the choice of priors and Markov chain Monte Carlo settings
☒	☐	For hierarchical and complex designs, identification of the appropriate level for tests and full reporting of outcomes
☐	☒	Estimates of effect sizes (e.g. Cohen's *d*, Pearson's *r*), indicating how they were calculated

Our web collection on statistics for biologists contains articles on many of the points above.

Software and code

Policy information about availability of computer code

Data collection	Next-generation sequencing data for SGE experiments were collected on an Illumina NovaSeq X instrument and demultiplexed using Illumina's bcl2fastq2.
Data analysis	All scripts are publicly available on GitHub: https://github.com/FrancisCrickInstitute/RNU4-2_Saturation_Genome_Editing.<br><br>Custom scripts written in Python v2.7.18 were adapted from published analyses (Buckley et al. 2024) and used to analyse NGS data and calculate function scores for SGE experiments, using the needle-all aligner from EMBOSS v6.6.0. To analyse data and generate figures, R v4.5.0 was used with RStudio v2025.05.1+513, and additional analyses were performed in Python v3.13.5. ViennaRNA v2.7.0 was used to predict binding energies of RNA structures. CADD v1.7 was used to generate CADD scores. DRAGEN v4.2 was used to identify variants in population cohorts. rMATS-turbo v4.3.0 was used for RNA-seq analysis.

For manuscripts utilizing custom algorithms or software that are central to the research but not yet described in published literature, software must be made available to editors and reviewers. We strongly encourage code deposition in a community repository (e.g. GitHub). See the Nature Portfolio guidelines for submitting code & software for further information.

Data

Policy information about availability of data

All manuscripts must include a data availability statement. This statement should provide the following information, where applicable:

- Accession codes, unique identifiers, or web links for publicly available datasets
- A description of any restrictions on data availability
- For clinical datasets or third party data, please ensure that the statement adheres to our policy

SGE data including all RNU4-2 function scores are available in Supplementary Table 1. Fastq files from SGE experiments are available on the European Nucleotide Archive (accession: PRJEB87505). RNA-sequencing data (Figure 3D) were taken from Nava et al. Nature Genetics 2025 and are available in the European Genome–Phenome Archive (EGA, http://www.ebi.ac.uk/ega; study accession EGAS50000000889). UK Biobank and All of Us V8 data are available to researchers upon approval of application (see https://www.ukbiobank.ac.uk/use-our-data/apply-for-access/ and https://www.researchallofus.org/).

Research involving human participants, their data, or biological material

Policy information about studies with human participants or human data. See also policy information about sex, gender (identity/presentation), and sexual orientation and race, ethnicity and racism.

Reporting on sex and gender	Human participants were included in analyses based solely on RNU4-2 genotype without regard for sex and gender.
Reporting on race, ethnicity, or other socially relevant groupings	We do not report race, ethnicity, or other socially relevant groupings.
Population characteristics	143 patients with pathogenic and likely pathogenic RNU4-2 variants and available phenotypic data were used to correlate SGE data to phenotypic severity. This pre-existing cohort is detailed in Nava et al. Nature Genetics (2025).<br><br>We identified UK Biobank participants with: (1) depleted variants in the 18 bp RNU4-2 CR (n = 6), (2) depleted variants outside of the CR (n = 50), and (3) participants with non-depleted SNVs outside of the CR (n = 12,132).<br><br>We searched rare disease cohorts for individuals with biallelic variants in RNU4-2. These cohorts included the Genomics England 100,000 Genomes Project and NHS Genomic Medicine Service datasets accessed through the UK National Genomic Research Library, the SeqOIA and Auragen clinical cohorts in France (PFMG 2025), the Undiagnosed Disease Network, the Broad Institute Center for Mendelian Genomics (CMG) and GREGoR (Genomics Research to Elucidate the Genetics of Rare Diseases) Consortium cohorts. We only included individuals with homozygous variants with function scores < -0.302, or compound heterozygous variants where both had function scores < -0.302 (n=20). All individuals had prior genome analysis including investigation of variants in known NDD genes and large structural variants. RNU4-2 genotypes for all 20 individuals are in Extended Data Table 3. Age at time of last follow-up ranged from <12 months (1 individual) to >18 years (6 individuals). A complete description of these and other population characteristics is provided in our companion manuscript (Ruis et al. 2026 Nature Genetics).
Recruitment	Participants were recruited to the Genomics England project and to rare disease cohorts based on clinical presentation. Other participants were recruited to studies including the UK Biobank and All of Us on a voluntary basis. Accordingly, the former studies are biased towards individuals with established clinical diagnoses while the later studies are biased towards healthy individuals.
Ethics oversight	Informed consent was obtained for all patients included in this study from their parent(s) or legal guardian. This study was approved by the 100,000 Genomes Project Protocol, which has ethical approval from the HRA Committee East of England Cambridge South (REC Ref 14/EE/1112). Each rare disease cohort used in this analysis previously received study approval by a local regulatory authority.<br><br>We received an exception to the Data and Statistics Dissemination Policy from the All of Us Resource Access Board to report questionnaire response data for the single individual with a homozygous depleted variant as well as variant counts < 20 for all variants in RNU4-2.

Note that full information on the approval of the study protocol must also be provided in the manuscript.

Field-specific reporting

Please select the one below that is the best fit for your research. If you are not sure, read the appropriate sections before making your selection.

☒ Life sciences ☐ Behavioural & social sciences ☐ Ecological, evolutionary & environmental sciences

For a reference copy of the document with all sections, see nature.com/documents/nr-reporting-summary-flat.pdf

Life sciences study design

All studies must disclose on these points even when the disclosure is negative.

Sample size	No sample size calculations were explicitly performed. For SGE experiments, variants to be tested were predefined in the process of library construction to include all pathogenic RNU4-2 variants known at time of library design, as well as all possible SNVs and 1-bp insertions and deletions in the RNU4-2 critical region. This resulted in an effective sample size of n = 539 variants to be tested by SGE. Established SGE protocols were used to ensure an adequate number of cells received each variant in the library to ensure reproducible scoring. This was confirmed by analysing the library distribution and score reproducibility in Figure 1c and Extended Data Figure 1.<br><br>For phenotypic analyses in relation to SGE function scores, all individuals with relevant genotypes were included from cohorts analysed. Individuals were retrospectively grouped based on genotype. The group of n= 20 individuals with biallelic SGE-depleted variants proved sufficiently large, as all 20 had an undiagnosed neurodevelopmental disorder.
Data exclusions	No data were excluded from analyses, however some analyses focus exclusively on SNVs rather insertions and deletions. This was because all possible SNVs were included in library design and assayed across the complete gene, whereas insertions and deletions were designed in a non-random fashion to regions with a higher or lower chance of being functionally impactful.
Replication	Experiments in this study comprised of three independent saturation genome editing experiments performed in both haploid and diploid HAP1 cells, meaning separate populations of cells were transfected, edited, cultured, and sequenced for each replicate. All variants were scored in each replicate, and reproducibility is plotted. Function scores were highly correlated (Figure 1c, Extended Data Figure 1c). No additional attempts at replication were performed. Data from individual replicates are available in Supplementary Table 1.
Randomization	Human genetic analysis comprised an observational study of genotypes and SGE function scores for variants in pre-existing cohorts, so randomization is not relevant. Instead, participants were allocated into groups based on RNU4-2 genotype.
Blinding	As SGE data is collected for a single pool of edited cells containing many variants, blinding is inherent to the experimental process. Human genetic analysis comprised an observational study of genotypes in pre-existing cohorts, so blinding is not relevant.

Reporting for specific materials, systems and methods

We require information from authors about some types of materials, experimental systems and methods used in many studies. Here, indicate whether each material, system or method listed is relevant to your study. If you are not sure if a list item applies to your research, read the appropriate section before selecting a response.

Materials & experimental systems

n/a	Involved in the study
☒	☐ Antibodies
☐	☒ Eukaryotic cell lines
☒	☐ Palaeontology and archaeology
☒	☐ Animals and other organisms
☒	☐ Clinical data
☒	☐ Dual use research of concern
☒	☐ Plants

Methods

n/a	Involved in the study
☒	☐ ChIP-seq
☒	☐ Flow cytometry
☒	☐ MRI-based neuroimaging

Eukaryotic cell lines

Policy information about cell lines and Sex and Gender in Research

Cell line source(s)	HAP1 cells were originally obtained from Haplogen, which is now Horizon Discovery.
Authentication	HAP1 stocks were previously validated by karyotyping, then later by staining for DNA content to assess and maintain ploidy.
Mycoplasma contamination	Mycoplasma testing was performed for HAP1 cells and confirmed negative.
Commonly misidentified lines<br>(See ICLAC register)	No commonly misidentified cell lines were used this study.

Plants

Seed stocks	*Report on the source of all seed stocks or other plant material used. If applicable, state the seed stock centre and catalogue number. If plant specimens were collected from the field, describe the collection location, date and sampling procedures.*
Novel plant genotypes	*Describe the methods by which all novel plant genotypes were produced. This includes those generated by transgenic approaches, gene editing, chemical/radiation-based mutagenesis and hybridization. For transgenic lines, describe the transformation method, the number of independent lines analyzed and the generation upon which experiments were performed. For gene-edited lines, describe the editor used, the endogenous sequence targeted for editing, the targeting guide RNA sequence (if applicable) and how the editor was applied.*
Authentication	*Describe any authentication procedures for each seed stock used or novel genotype generated. Describe any experiments used to assess the effect of a mutation and, where applicable, how potential secondary effects (e.g. second site T-DNA insertions, mosiacism, off-target gene editing) were examined.*
