# Supplementary material for: Saturation editing of RNU4-2 reveals distinct dominant and recessive disorders
Source: Nature. 2026 Apr 8;654(8118):429–36. doi: 10.1038/s41586-026-10334-9 (PMC13253345; doi:10.1038/s41586-026-10334-9)
Supplement: Supplementary file 3 — List of variants in RNU4ATAC in ClinVar. [file 41586_2026_10334_MOESM3_ESM.pdf]

U4atac HGVS	Chr	Pos	ClinVar variation ID	ClinVar classification	U4 nt	Matching ref base	Function score	Category	Region
n.8C>A	2	121530887	692040	Likely pathogenic					Stem II
n.13C>T	2	121530892	218083	Pathogenic					Stem II
n.16G>A	2	121530895	218082	Pathogenic/Likely pathogenic					Stem II
n.29T>G	2	121530908	977869	Likely pathogenic					5' stem loop
n.30G>A	2	121530909	30180	Likely pathogenic					5' stem loop
n.35A>C	2	121530914	2674599	Likely pathogenic	30	Y	-0.617	UKB/AllofUs	5' stem loop / k-turn
n.37G>A	2	121530916	218084	Pathogenic/Likely pathogenic	32	Y	-1.267	UKB/AllofUs	5' stem loop / k-turn
n.40C>T	2	121530919	599282	Pathogenic	35	N			5' stem loop / k-turn
n.46G>T	2	121530925	977856	Likely pathogenic	41	N			5' stem loop / k-turn
n.46G>A	2	121530925	636959	Pathogenic	41	N			5' stem loop / k-turn
n.48G>A	2	121530927	218085	Pathogenic/Likely pathogenic	43	Y	-0.173	UKB/AllofUs	5' stem loop / k-turn
n.50G>C	2	121530929	30182	Likely pathogenic	45	Y	-0.864	UKB/AllofUs	5' stem loop / k-turn
n.51G>A	2	121530930	30178	Pathogenic/Likely pathogenic	46	Y	-0.507	UKB/AllofUs	5' stem loop / k-turn
n.53C>G	2	121530932	30183	Pathogenic/Likely pathogenic	48	N			5' stem loop
n.55G>A	2	121530934	30179	Pathogenic	50	Y	-0.331	UKB/AllofUs	5' stem loop
n.111G>A	2	121530990	30181	Pathogenic					3' stem loop
n.114G>C	2	121530993	977780	Pathogenic	116	Y	-0.154	unobserved	3' stem loop
n.116A>C	2	121530995	1373455	Pathogenic	118	Y	-0.814	UKB/AllofUs	Sm protein binding
n.116A>G	2	121530995	870579	Likely pathogenic	118	Y	-0.872	UKB/AllofUs	Sm protein binding
n.116A>T	2	121530995	812960	Likely pathogenic	118	Y	-0.169	UKB/AllofUs	Sm protein binding
n.117A>G	2	121530996	1525441	Pathogenic	119	Y	-0.725	UKB/AllofUs	Sm protein binding
n.120T>G	2	121530999	977779	Pathogenic	122	Y	-0.737	unobserved	Sm protein binding
n.124delG	2	121531002	1224468	Likely pathogenic	126	N			Sm protein binding
n.124G>T	2	121531003	2813456	Pathogenic	126	N			Sm protein binding
n.124G>A	2	121531003	39443	Pathogenic/Likely pathogenic	126	N			Sm protein binding
n.23C>T	2	121530902	1133987	Likely benign					loop region
n.41G>A	2	121530920	1629605	Likely benign	36	N			loop region
n.42C>T	2	121530921	1160662	Likely benign	37	N			loop region
n.43A>G	2	121530922	1633266	Likely benign	38	N			loop region
n.45A>C	2	121530924	1632468	Likely benign	40	N			loop region
n.58C>T	2	121530937	1170904	Benign					loop region
n.65C>T	2	121530944	1631270	Likely benign					Stem I
n.87C>T	2	121530966	1168718	Benign/Likely benign					3' stem loop
n.93G>A	2	121530972	1601357	Benign					3' stem loop
n.109T>A	2	121530988	1628512	Likely benign					3' stem loop
n.117dup	2	121530994	1159385	Likely benign					Sm protein binding

Supplementary Table 2. List of variants in *RNU4ATAC* in ClinVar. The equivalent residue of *RNU4-2* was determined for variants in the 5’ stem loop / k-turn and Sm protein binding site (see **Methods**). Function scores are included where an equivalent nucleotide could be determined and the reference base at that position is the same across *RNU4-2* and *RNU4ATAC*.
