# Supplementary material for: Saturation editing of RNU4-2 reveals distinct dominant and recessive disorders
Source: Nature. 2026 Apr 8;654(8118):429–36. doi: 10.1038/s41586-026-10334-9 (PMC13253345; doi:10.1038/s41586-026-10334-9)
Supplement: Supplementary file 5 — Peer Review File [file 41586_2026_10334_MOESM5_ESM.pdf]

nature portfolio

Peer Review File

Saturation editing of *RNU4-2* reveals distinct dominant and recessive disorders

Corresponding Author: Dr Nicola Whiffin

Any redactions in this file are there to maintain patient confidentiality, the confidentiality of unpublished data, or to remove third-party material.

This file contains all reviewer reports in order by version, followed by all author rebuttals in order by version.

Version 1:

Reviewer comments:

Referee #1

(Remarks to the Author)
Summary of the key results
The authors of this manuscript conducted saturation genome editing of RNU4-2, a small nuclear RNA that was recently discovered to cause the prevalent neurodevelopmental disorder ReNU syndrome. By generating a variant effect map spanning the length of RNU4-2, the authors of this manuscript present several key findings including identification of a novel recessive NDD caused by distinct variants in RNU4-2, redefinition of the critical 18bp region where de novo variants are associated with ReNU syndrome into two shorter regions, and correlation of clinical severity of ReNU syndrome with the degree of variant effect as measured by SGE. These data demonstrate the clinical importance of empirically measuring noncoding variant function, as computational variant effect predictors perform poorly in the noncoding space.

Originality and significance:
The manuscript, which utilises well established existing methods in a largely robust manner to generate high quality and well-presented data, communicates findings likely to be of immediate interest to the rare-disease and clinical genetics communities, in addition to those interested in spliceosome and non-coding RNA biology, and developing and performing multiplexed assays of variant effect (MAVE). The data are likely to be of immediate practical utility in clinical variant classification. However, the experimental technique used is not novel, and the manuscript would benefit substantially from extending the experimental work.

Data and methodology:
Overall, this is a valid approach, the data are of high quality and are well presented. Specific suggested improvements are listed below.

Appropriate use of statistics and treatment of uncertainties:
1. The saturation genome editing experiments in this manuscript only comprise of two replicates. While there is good correlation seen between these two replicates, I would consider three replicates to be the minimum required for robust statistical analysis. I would strongly suggest that a third biological replicate is performed. This is particularly important because the data are likely to be used immediately by the clinical community in diagnostic classification, and therefore all reasonable efforts need to be taken to make them as robust as possible.

2. Please provide more clarity on how normalisation to control variants was performed. Was this by subtracting the median score of the control variants? Why was this performed twice (both before and after averaging across replicates)? I would suggest normalising only once to control variants.

3. Would a non-linear method like UMAP be more useful than PCA for clustering of ReNU syndrome cases by phenotype?

Suggested improvements:
Major comments:
Results:

1. This manuscript refers to 16 individuals with biallelic variants in RNU4-2, whereas the companion paper on the recessive condition refers to 33 individuals. I don't understand why this smaller subset is used in the SGE paper. Reading the companion manuscript, it becomes clear that only 14/30 of these variants significantly deplete in the SGE assay. This is important, as incorrect calibration of the SGE data using only the subset of variants reported in this paper would result in incorrect weighting of these data for use in clinical variant classification.
2. I would strongly suggest that the two papers are merged. As it stands there is substantial repetition, and it is confusing that in the SGE paper only a subset of recessive variants are reported. It is also of relevance to the clinical community that SGE effect scores correlate with clinical phenotypic severity.
3. I appreciate that there are differences between the clinical presentations of the recessive and dominant RNU4-2 associated disorders. However, there is also substantial overlap between the conditions, and it is likely that patients will present with single variants and a phenotype that could fit either condition. An important innovation in the field would be to apply saturation genome editing in a diploid cell line, and to directly test whether variants have a dominant or recessive effect. I would strongly recommend that the authors attempt to perform functional evaluation of RNU4-2 variants in an alternative cell line to see whether they can better differentiate between recessive and dominant variants. The RNU4-2 locus is very small, and the size of the HDR library used is modest (539 variants), which makes such an experiment manageable, and would substantially increase both the novelty and clinical utility of the results. In my opinion, this experiment would significantly advance the field and make this paper highly novel, fitting for publication in Nature.
4. For clinicians to use these data in diagnostic variant classification they need to be calibrated such that a strength of evidence can be applied within the ACMG framework. While I appreciate that this framework may change in the near future, it is important to calibrate the variants for use now. I would therefore suggest that the authors follow the recommendations in Brnich et al. 2020 (Genome Medicine) to calibrate these data for clinical use.
5. The Chan et al 2024 paper included RNA sequencing of patients with ReNU syndrome. It would be very useful if the authors could carry out RNA sequencing of patients with the recessive syndrome associated with RNU4-2 and compare this with the ReNU syndrome data. This may identify clinically useful biomarkers that will facilitate further resolution of VUS.

Minor Comments:
Introduction
1. The authors state that they “developed a strategy” to combat the high sequence homology of RNU4-2 with its homologs and pseudogenes. This is strong phrasing for a minor modification of an established approach – perhaps “identified a method” or “implemented an approach” would be more appropriate.

Methods:
1. Please present details of experimental QC of the HDR library using a high-throughput sequencing method to assess even representation of all designed variants, detect synthesis bias or missing/underrepresented variants, and quantify the proportion of wildtype and PAM-only sequences.
2. The authors state that all but one variant known to cause NDD were included in the assay (line 110), but do not state the reason for this exclusion, or why some VUS were not tested (presumably they were reported after the HDR library was designed?).
3. Include a more detailed description of how the PAM-disrupting mutation was selected (e.g. what is the corresponding CADD/PhyloP score of the position of this mutation?).
4. Please clarify what the allele count threshold used to define ‘population negative control variants’ is, and why it was chosen.

Results:
1. 539 variants is a modestly size SGE library. Given the mutational rate in this region, this is somewhat of a missed opportunity to have not tested indel insertion along the length of the RNU4-2 transcript.
2. Define the “six distinct regions where variants score as depleted” mentioned (line 125) in genomic coordinates.
3. It would be useful to compare the SGE data to in-silico variant effect prediction tools other than CADD. For example, are there popEVE scores available for RNU4-2?
4. How does the projected prevalence of the recessive NDD associated with RNU4-2 compare to that of ReNU syndrome? This information is in the accompanying clinical manuscript but should be included in the SGE paper also.
5. Figure 1C/D. It is not immediately apparent that the figure legend for Figure 1D also applies to 1D. Either put C and D on the same row and keep the one figure legend. Alternatively, add a comment in the figure legend that the legend for Figure1C is the same as that for Figure 1D.
6. Figure 1D. It would be useful to highlight visually in the figure the “six distinct regions where variants scored as depleted” mentioned in the results before the authors reference Figure1D. For example, it is unclear where the (assumed) right-most region finishes when interpreting this by eye. Clinically it is important to know what the boundaries of these regions are in genomic coordinates.
7. Figure 1A/C/D. The purple colour used for multi-nt insertions is hard to see. Could a more contrasting colour be chosen?
8. Figure 2D. It’s confusing that there are two variants coloured in blue, which have an AC=0 in UKBB. Presumably, these are variants that were seen in the All of Us cohort. Would it be possible to plot instead the sum of the allele counts in both cohorts against the SGE score?
9. Figure 3b. It would be useful to give the number of patients phenotyped for each category above the plot (ie document n=x above the labels “ID, “GDD”, “speech” and “seizures”. This will be a useful guide to clinicians if they see a patient who does not fit this pattern.
10. Figure 4. Shaded teal regions are a very similar colour to that of neutral SNVs. Could a more contrasting colour be used?
11. Figure 4. “Black” triangles appear grey. Please change the colour or the legend description for clarity.
12. Supplementary Figure 1: it is not clear to my why just 12 ReNu syndrome variants were included, why not all known dominant variants?

13. Supplementary Figure 1: Differences in dot sizes indicating number of variances are subtle. Contour lines or plot labels showing exact variant counts for key regions might help interpretation.
14. Supplementary Figure 2. Include a description of the vertical red dashed lines.
15. Thank you for providing all of the data as a clearly laid-out supplementary file. Please could you include reference transcript ID within the HGVS ID given?

Discussion:
1. Where the authors list "ascertainment bias, germline selection, and/or an elevated mutation rate" (line 48) as possible explanations for the high recurrence of the single nucleotide insertion (n.64_65insT) observed in individuals with ReNU syndrome, the discussion only addresses ascertainment bias. I think it would be appropriate to briefly reiterate and cite the discussion of these hypotheses made in the Chen, Y. et al. 2024 in the discussion of this paper, or explain why ascertainment bias is thought to be the most likely explanation.

References:
Appropriate papers are cited throughout to give an overview of the existing state of the field. However, Greene et al. 2024 reported that the two distinct regions were associated with ReNU syndrome causing variants: (n.62–70 and n.73–79). Although the newly defined 4-nt region (n.75-78) does not exactly match that of Greene et al. 2024, the 9-nt region (n.62-70) was correctly identified by Greene et al, and it would seem reasonable to acknowledge this.

Clarity and context:
The manuscript is clearly written and logically structured. The abstract is concise and clearly lays out the authors' intent, approach, key findings, and significance of this work. The introduction is well-grounded in prior literature and succeeds in contextualising readers to existing knowledge and knowledge gaps, as well as relevant experimental work which justifies this research.

In conclusion: this is a well conducted study that will be of interest to a wide readership. If the improvements suggested above are made, I would recommend it's publication in Nature.

(Remarks on code availability)
A README file is provided with sufficient instructions for installing and running the code. The code is well annotated and easy to read.

Referee #2

(Remarks to the Author)
I co-reviewed this manuscript with one of the reviewers who provided the listed reports.

(Remarks on code availability)
Code is publicly accessible and scripts are well annotated, with a corresponding README file in the specified GitHub repository. We as reviewers have not yet had the opportunity to completely install and re-run the code accompanying this manuscript and therefore haven't included commentary on this aspect.

Referee #3

(Remarks to the Author)
In this manuscript, De Jonghe and collaborators perform saturation genome editing (SGE) of RNU4-2, which encodes U4 snRNA, an essential component of the major spliceosome. The authors and another group recently reported that variants in RNU4-2 cause the neurodevelopmental disorder (NDD) ReNU syndrome. Here, the authors adapted an SGE protocol for the repetitive RNU4-2 locus and tested the effect of 539 RNU4-2 variants on cell growth, which allowed them to compute a function score. They show that this function score accurately discriminates between variants known to cause ReNU syndrome and variants present in population databases. They narrow the critical region associated with ReNU syndrome and show correlation between function scores and NDD severity. They also identify variants with significant function scores in other regions of RNU4-2 and report that these are associated with a new recessive NDD distinct from ReNU syndrome, for which the phenotype is described in a companion paper.

The data presented are interesting, novel, and would introduce a much-needed approach to evaluate the pathogenicity of variants in non-coding RNAs. The manuscript is clear and well-written. I believe that the manuscript would represent an important addition to the literature. However, the conclusions and significance of the study would be enhanced by linking the SGE function score with the function of U4 snRNA in splicing, as well as adding a few clarifications and analyses, as detailed below.

- The SGE function score is solely based on a cell growth readout. Yet, given the key role of U4 snRNA in splicing, how the function score relates to splicing should at least be investigated for a subset of variants (e.g. selected ReNU vs. UKB/AllofUs vs. unobserved variants in different regions, variants with strong vs. moderate function scores). For example, selected variants could be introduced in cells followed by RNA-seq and/or using a targeted method to assess splicing events previously linked with ReNU. Pooling variants that were shown to have normal, moderate or strong function scores, and

comparing splicing profiles between these pools could also be done. Such experimental validation would significantly strengthen the impact of the study and validate the use of a cell growth readout for assessing the pathogenicity of snRNA variants. Alternatively, the authors previously reported (Nava et al. Nat Genet 2025) that ReNU patients show altered 5'SS usage in lymphoblasts and that splicing profiles can discriminate between mild and severe cases. The authors could at least show whether "strong" and "moderate" function scores correlate with the extent of splicing alterations in those patients.

- Related to the previous point, the authors show that neighboring variants within the ReNU critical region differ in their function score, including variants with normal, moderate and strong function scores. Do the function scores correlate with predicted impacts on U4 secondary structure and/or base pairing with U6 snRNA (e.g. using RNA structure prediction software)?

- While the overall correlation between replicates is good, some positions show more variability between replicates, including some ReNU variants that are just below the function score threshold when considering the average between replicates, but with one replicate above threshold. Variability between replicates should be considered when assigning variants to moderate or strong categories.

- In Fig. 3B, do the trends hold if separated by variants? Are there specific variants that drive this effect?

Minor comments:

- The use of the HAP1 cell line should be justified in the text when it is first introduced. While its haploid nature facilitates SGE, it is a distinct cellular type from those affected in NDD. Similarly, the authors should justify why using cell growth as a readout for NDD pathogenicity is appropriate.

- Fig. 3B: what the error bars represent should be specified (between variants?). A binomial test with default features (p=0.5) doesn't seem appropriate to obtain 95% confidence intervals, given that there are three phenotypic categories for each function score category.

(Remarks on code availability)
I have reviewed the secondary code and was able to run it with minor modifications (e.g. changing the name of the file RNU4_2_ST1_15042025.csv and skipping the header line). In the Python code, additional comments to explain each filter would be useful.

Referee #4

(Remarks to the Author)
This study builds upon foundational work from this team (Chen et al 2024, Nature) and others (Greene et al 2024, Nature Medicine) who first reported de novo pathogenic variants in RNU4-2, a non-coding RNA component of the spliceosome, as a major cause for a neurodevelopmental disorder (NDD) termed ReNU syndrome. Given the prevalence of the condition, and suboptimal performance of in silico algorithms (i.e. CADD) at predicting pathogenicity, there is a major need to experimentally determine which variants within the RNU4-2 locus are pathogenic and whether a phenotype correlation can be established by variant or region of the ncRNA that is impacted. The current study tackles these gaps through a saturated genome editing (SGE) approach using HAP1 cells: (a) to systematically identify the functional impacts of variants across RNU4-2 and (b) to delineate variants by phenotypic severity. In the process, they identify variants outside the previously established RNU4-2 critical region that cause an NDD that differs from ReNU syndrome in both its inheritance pattern (recessive), and its clinical sequelae (particularly MRI findings; reported in a separate companion paper by Rius and colleagues). This elegant study illustrates the power of SGE to assign variant pathogenicity at a resolution sufficient to define critical regions of RNU4-2 not possible with genetics or in silico predictions alone. The controls for the SGE are robust, including all but one known disease-associated variant as positive controls (including the recurrent n.64_65insT change); and as negative controls, twelve 1-nt insertions in stem loops outside the CR that were not predicted to be deleterious. The output of the study is impactful given the prevalence of the disorder and effectiveness of SGE. This reviewer anticipates that this study will be a heavily used resource and hopes that it will serve as a springboard for iterative updates as new RNU4-2 cases and variants (if not studied here, i.e. indels) are reported in the future. However, some lingering questions remain that warrant further attention prior to publication:

Major points:
1. ReNU is not a new disease entity nor is SGE a new technique. Thus, the major novelty of the paper is twofold: (1) a repertoire of variants and predicted pathogenicity, and (2) a hitherto unreported recessive NDD caused by RNU4-2. The latter left me wanting much more in terms of answering the question of why phenotypes are clinically distinct from ReNU. Neither manuscript begins to answer this question sufficiently or speculates to satisfaction. RNAseq should be performed on cell lines from affected individuals with the recessive RNU4-2 NDD for comparison with the ReNU samples reported by Chen et al. Even if access to samples from affected individuals is not possible, the authors have demonstrated their expertise with CRISPR/Cas9 and could edit these regions of the gene to simulate biallelic variants so that global impacts on splicing can be determined. This key experiment would offer a first glimpse of the molecular differences between the two RNU4-2 disorders, which is much needed to meet the bar for publication in Nature.

Minor points

1. The authors mention the challenges to specifically target RNU4-2 due to high sequence homology with RNU4-1. Did they confirm specificity by sequencing RNU4-1 in the HAP-1 cells used for SGE? A brief mention in the main text would offer reassurance.

2. Although the eight multinucleotide insertions in the CR that were included as positive controls were all depleted (functional scores well below the -0.39 threshold), it is intriguing that not all the 1-nt insertions were scored as pathogenic (Fig 1D, especially in Stem III). Can the authors explain why this might be?

3. Can the authors please confirm that in the individuals with recessive RNU4-2 NDD that there were no other candidate causal genes or variants elsewhere in the genome; particularly any other spliceosome related ncRNA or CNVs. This may be more appropriate to include in the companion paper but deserves a mention.

4. The discussion paragraph beginning on line 347 states “we cannot exclude the possibility that variants with more subtle effects may be clinically relevant”. It would make sense to also state the following (or something similar) “nor can we exclude the possibility that variants that score at or near the -0.39 function score threshold are benign, potentially representing false positives that should be interpreted with caution, particularly when clinical decisions are at stake”

5. Supplementary Table 1 would benefit from a column that has the calculated mean function score. Although any reader could do this themselves, it would make it easier to cross-reference specific variants highlighted in the main text and figures.

6. The Figure 4 legend mentions black triangles corresponding to homologous positions of RNU4ATAC. These triangles appear to be gray in the figure; please modify for consistency.

7. A minor cosmetic point, but the horizontal dashed line in Figure 2A is barely visible. Changing the shade or color would assist readers.

(Remarks on code availability)
The source code for the primary and secondary analysis code are available, as are the raw sequencing data. The code appears to be a usable resource for the community.

Version 2:

Reviewer comments:

Referee #1

(Remarks to the Author)
Summary of the key results
The authors of this manuscript conducted saturation genome editing of RNU4-2, a small nuclear RNA that was recently discovered to cause the prevalent neurodevelopmental disorder ReNU syndrome. By generating a variant effect map spanning the length of RNU4-2, the authors of this manuscript present several key findings including identification of a novel recessive NDD caused by distinct variants in RNU4-2, redefinition of the critical 18bp region where de novo variants are associated with ReNU syndrome originally identified in the previous publication by Chen et. al., and correlation of clinical severity of ReNU syndrome with the degree of variant effect as measured by SGE. These data demonstrate the clinical importance of empirically measuring noncoding variant function, as computational variant effect predictors perform poorly in the noncoding space.

Originality and significance:
The manuscript, which utilises well established existing methods in a robust manner to generate high quality and well-presented data, communicates findings likely to be of immediate interest to the rare-disease and clinical genetics communities, in addition to those interested in the biology of non-coding RNAs, and developing and performing multiplexed assays of variant effect (MAVE). The data are likely to be of immediate practical utility in clinical variant classification. Though the experimental technique used is not novel, this manuscript presents experimentally sound data strengthened by the authors’ revisions.

Data and methodology:
This is a valid approach, the data are of high quality and are well presented. The authors’ revisions have largely resolved our concerns.
The comparison of RNA sequencing data of patients with the recessive syndrome associated with RNU4-2 and ReNU syndrome is a very welcome addition. The identification of a distinct expression pattern and potential biomarker is a clinically important discovery. The discussion of this manuscript adequately summarises these findings and we find the full presentation of these analyses in the companion manuscript to be appropriate to limit redundancy between the two.

Appropriate use of statistics and treatment of uncertainties:
We thank the authors for performing a third biological replicate, this has strengthened their findings.

The authors have clarified their normalisation method and provided a rationale for their approach. While we remain uncertain that normalising the data twice is required, the added Methods text now clarifies describes their chosen two-step

normalisation procedure, and this is unlikely to substantially change their findings.

Conclusions:
The authors' calibration of SGE function scores is well implemented and consistent with recommendations in Brnich et al. In the absence of benign variants reported in ClinVar, we regard the use of a combined allele count >100 between UK Biobank and All of US as a sensible substitution.
We appreciate the authors caution in not calibrating the assay for variants outside of the ReNU CR. However, in practice, clinicians will use these data to assess variants in this region, and will look to these two publications for guidance in how to use these data in clinical variant classification. How do the authors suggest the SGE data be used in regions outside of the ReNU CR? we presume the authors recommend ascribing 'Supporting evidence' if a variant has a statistically significant SGE score (pending calibration of SGE scores using an independently-ascertained cohort)? We think describing this clearly in the discussion is important (in both manuscripts).

It is not currently sufficiently clear in this manuscript that not all individuals with biallelic RNU4-2 variants have statistically significant SGE results. For example: the following additions would be helpful:
- On page 11: "The clinical phenotypes of the twenty identified NDD individuals are characterised as part of a broader cohort (total n = 38) in a companion manuscript. The 18 additional individuals reported in this broader cohort have biallelic RNU4-2 variants with non-significant functional scores".
-On page 12: "This NDD is described comprehensively in a companion manuscript, in which we also expand the cohort to include 38 individuals: the 20 individuals with biallelic variants with significant function scores that are presented here, and 18 additional individuals with variants that are not significantly SGE-depleted, but are found in the same functional regions of RNU4-2."

References are appropriate throughout

Clarity and context:
The manuscript is clearly written and logically structured. The abstract is concise and clearly lays out the authors' intent, approach, key findings, and significance of this work. The introduction is well-grounded in prior literature and succeeds in contextualising readers to existing knowledge and knowledge gaps, as well as relevant experimental work which justifies this research.

In conclusion: this is a well conducted study that will be of interest to a wide readership. We recommend the publication of this manuscript in Nature.

(Remarks on code availability)
The code is well annotated, and is a usable resource for the community.

Referee #2

(Remarks to the Author)
I co-reviewed this manuscript with one of the reviewers who provided the listed reports.

(Remarks on code availability)
The code is well annotated, and is a usable resource for the community.

Referee #3

(Remarks to the Author)
I thank the authors for addressing my major concerns. I believe the manuscript is significantly improved, notably by showing an association between SGE score and splicing disruption, and is now suitable for publication if the two minor comments below are addressed.

- Fig. 6A of Nava et al. reports 19 patients and 21 controls, whereas Fig. 3B of this manuscript used 19 patients and 20 controls, resulting in slight changes to the PCA. While the results may not change substantially, it would be more transparent to indicate why one control was removed, which apparently changes the number of significant A5SS events from 111 to 101.

- For the RNU4-1 and RNU4-2 expression analysis in the companion paper (but mentioned in the Discussion of this manuscript), the two transcripts have >97% sequence identity. Are the 4 mistmaches sufficient for the majority of reads to be uniquely mapped to one of the two transcripts in the DRAGEN pipeline? Otherwise, how were reads mapping to both transcripts handled?

In addition, while I understand the authors' rationale for splitting the results into two papers, I agree with Reviewer #1 that merging the two papers would increase clarity and decrease repetition, especially for key results that are relevant to both papers (e.g. RNA-seq analysis comparing ReNU and recessive NDD).

(Remarks on code availability)
I did not review the code again, as it was satisfactory in the first submission.

Referee #4

(Remarks to the Author)
A. Summary
This article and its companion paper under review in Nature Genetics have been substantially bolstered by new data, and both are markedly improved. The main changes to the manuscript are as follows: (1) addition of a third replicate SGE, (2) authors performed SGE in diploid cells; (3) calibrated function scores to evidence strengths for clinical classification; (4) correlated SGE scores with predicted changes to RNA structure; (5) added an analysis to correlate SGE scores with extent of splicing disruption seen in ReNU patients; and (6) identified additional patients with recessive RNU4-2 NDD.

B. Originality
This elegant study illustrates the power of SGE to assign variant pathogenicity at a resolution sufficient to define critical regions of RNU4-2 not possible with genetics or in silico predictions alone.

C. Data and methodology
The data are robust and presentation is clear.

D. Statistics
No concerns.

E. Conclusions
The conclusions are robust and well-supported with appropriate positive and negative controls.

F. Suggested improvements
These main changes address my previous major comments, and the authors have responded to my prior minor concerns to satisfaction. I am pleased with the content and overall message of this manuscript and support acceptance for publication in Nature with the provision that the two following minor comments be addressed:

1. Supplementary Table 1: Please include definitions of abbreviations, especially for information in the headers.

2. RNA-sequencing: There is RNA-seq cluster analysis described in the Methods and data shown in Fig 3D (19 ReNU samples and 20 controls or the PCA of the PSI values). Please ensure that the RNAseq data are publicly available and indicate how to access datasets in the Data Availability section.

G. References
No concerns; citations are appropriate

H. Clarity
The manuscript is clear for a broad readership and will hopefully be used as a helpful resource for clinicians, basic scientist, and anyone interested in furthering knowledge on RNA splicing in development and disease.

(Remarks on code availability)

Open Access This Peer Review File is licensed under a Creative Commons Attribution 4.0 International License, which permits use, sharing, adaptation, distribution and reproduction in any medium or format, as long as you give appropriate credit to the original author(s) and the source, provide a link to the Creative Commons license, and indicate if changes were made.
In cases where reviewers are anonymous, credit should be given to 'Anonymous Referee' and the source.
The images or other third party material in this Peer Review File are included in the article's Creative Commons license, unless indicated otherwise in a credit line to the material. If material is not included in the article's Creative Commons license and your intended use is not permitted by statutory regulation or exceeds the permitted use, you will need to obtain permission directly from the copyright holder.
To view a copy of this license, visit https://creativecommons.org/licenses/by/4.0/

Response to Reviewers for "Saturation genome editing of RNU4-2 reveals distinct dominant and recessive neurodevelopmental disorders" by De Jonghe *et al.*

We would like to thank the reviewers for their positive and constructive comments on our manuscript. We have made changes in this revision to address their concerns and believe the manuscript has been improved as a result. A summary of the main changes is followed by a full point-by-point response to the reviewers' comments below.

A summary of the main changes is as follows:

1. We have performed a third replicate SGE experiment to enhance data quality and have updated all scores and statistics accordingly. This enabled us to refine the threshold used to classify variants as significant to -0.302.
2. We performed SGE experiments (in triplicate) in diploid cells to assess suitability for deciphering dominant and recessive variants *in vivo*, as requested by Reviewer 1. This experiment revealed variant effects to be attenuated across the gene, with the exception of previously depleted variants in Stem III, which all scored neutrally in diploid cells (**Sup. Fig. 7**).
3. We have calibrated function scores to evidence strengths for clinical classification of variants in accordance with established guidelines, as suggested by Reviewer 1 (**Sup. Fig. 5**).
4. We have included an analysis of correlation of the SGE scores with predicted changes to RNA structure using ViennaRNA (**Sup. Fig. 3**).
5. We have added an analysis to show that our SGE scores correlate with the extent of splicing disruption seen in ReNU patients (**Fig. 3D**).
6. We have identified additional patients with the recessive NDD: we now include 20 individuals who have two alleles with significant SGE scores in **Fig. 4**.

We have also substantially updated our companion manuscript, which now includes a characterised cohort of 38 recessive patients (including the 20 detailed here). Of particular note is addition of RNA-sequencing data analysis from individuals with biallelic *RNU4-2* variants and comparison of these data to that from individuals with ReNU syndrome. The results of this analysis are included in an **Appendix** at the end of this document.

Our responses below are in blue, with revisions to the manuscript quoted in pink.

Reviewer #1

Appropriate use of statistics and treatment of uncertainties:
1. The saturation genome editing experiments in this manuscript only comprise of two replicates. While there is good correlation seen between these two replicates, I would consider three replicates to be the minimum required for robust statistical analysis. I would strongly suggest that a third biological replicate is performed. This is particularly important because the data are likely to be used immediately by the clinical community in diagnostic classification, and therefore all reasonable efforts need to be taken to make them as robust as possible.

Thank you for this suggestion. We have now performed a third biological replicate and adjusted the analyses, figures, and tables throughout the manuscript as a result. This third replicate enables us to further refine our significance threshold to -0.302 with 151 variants now reported as significantly depleted compared to 138 previously. None of the main conclusions or results in the manuscript change as a result.

2. Please provide more clarity on how normalisation to control variants was performed. Was this by subtracting the median score of the control variants? Why was this performed twice (both before and after averaging across replicates)? I would suggest normalising only once to control variants.

You are correct in that the median score (log2-scale) of control variants was subtracted to perform the normalisation. This was first performed for individual replicates to facilitate comparison to one another (as in **Fig. 1C**). This ensures controls in both replicates are anchored on a median of 0.0. The final function scores provided (now, mean scores from three biological replicates) are normalised a second time to ensure the median final score of controls also equals 0.0 (NB: this adjustment is very small due to the first normalization having already been performed). Given the linear nature of the normalisation, final function scores reported would be equivalent if a single normalisation was performed only after averaging scores across replicates, but we think each replicate should be normalised in the same fashion to facilitate comparison across replicates.

We have provided more clarity on this procedure in the revised section of the Methods: "Function scores for library variants were first calculated per replicate, computed as the log_2-ratio of day 14 to day 4 variant frequencies, normalised by subtracting the median function score of negative control insertions from all scores. Final function scores were then calculated for each variant by averaging function scores across replicates, again normalising to the median of negative control insertions such that the median final function score of control insertions equals 0."

3. Would a non-linear method like UMAP be more useful than PCA for clustering of ReNU syndrome cases by phenotype?

We investigated using a UMAP approach for phenotype clustering and the output looks very similar to that from the PCA. This additional plot is now included as **Sup. Fig. 6B**.

Major comments:
Results:
1. This manuscript refers to 16 individuals with biallelic variants in RNU4-2, whereas the companion paper on the recessive condition refers to 33 individuals. I don't understand why this smaller subset is used in the SGE paper. Reading the companion manuscript, it becomes clear that only 14/30 of these variants significantly deplete in the SGE assay. This is important, as incorrect calibration of the SGE data using only the subset of variants reported in this paper would result in incorrect weighting of these data for use in clinical variant classification.

We intended for the two papers to be aligned, but this was difficult while we were still recruiting patients (and phenotype information) for the more clinically focussed paper. In the

clinical paper we now characterise a total of 38 individuals, with biallelic variants in *RNU4-2*. Of these 38 individuals, 20 have variants with significant SGE scores on both alleles. Because the interpretation of the additional cases relies on the clinical characterisation in the companion manuscript, we still only include these 'SGE significant' cases in the results of this current paper, however, we now mention the additional cases in the discussion. Specifically, we include: "This NDD is described comprehensively in a companion manuscript[22], in which we also expand the cohort to include 38 individuals: the 20 individuals with biallelic variants with significant function scores that are presented here, and 18 additional individuals with variants in the same functional regions of *RNU4-2*."

While we appreciate the importance of carefully calibrating functional assays for variant classification, the conservative threshold we set to identify significantly depleted variants was sufficient to discover the new recessive disorder, which is what is fundamental to this story's importance. We cannot yet calibrate these data for use in diagnosing the recessive disorder per ACMG guidelines as we do not have a suitable dataset of known pathogenic and benign variants to do so given that these two papers provide the first description of the disorder. Indeed, Brnich et al. explicitly discourage such a calibration as it would be circular in nature. We do now calibrate function scores for critical region variants for diagnosing ReNU syndrome, as described below. We include this point in our discussion: "While we showed that function scores for variants within the ReNU CR can provide strong evidence for clinical interpretation, we were unable to calibrate our assay for variants outside the ReNU CR due to a lack of independently defined pathogenic variants in these regions[8], as all individuals with recessive NDD were identified on the basis of function score."

2. I would strongly suggest that the two papers are merged. As it stands there is substantial repetition, and it is confusing that in the SGE paper only a subset of recessive variants are reported. It is also of relevance to the clinical community that SGE effect scores correlate with clinical phenotypic severity.

We have discussed this with the editor and with their support have chosen to keep these papers separate. We believe that the clinical data would get lost within all of the data in the SGE manuscript, even more so with the additional data and analysis included in this revision, and would not be visible enough to the target audience. The companion manuscript includes in-depth clinical characterisation of the recessive NDD through a substantial cohort of patients. It also includes comparison to ReNU syndrome at the genetic, phenotypic and now mechanistic levels. The latter, through addition of analysis of RNA-sequencing data from individuals with the recessive NDD and comparison to ReNU syndrome (see **Appendix**), further differentiates the two papers. We agree that the results of the SGE are of high relevance to the clinical community, which is why we have written the two manuscripts as companions rather than entirely separate papers. Nevertheless, we have made edits to both papers to try to reduce repetition and have clarified the difference in the patient numbers, both as detailed above here, and in the companion manuscript.

3. I appreciate that there are differences between the clinical presentations of the recessive and dominant RNU4-2 associated disorders. However, there is also substantial overlap between the conditions, and it is likely that patients will present with single variants and a phenotype that could fit either condition. An important innovation in the field would be to apply saturation genome editing in a diploid cell line, and to directly test whether variants

have a dominant or recessive effect. I would strongly recommend that the authors attempt to perform functional evaluation of RNU4-2 variants in an alternative cell line to see whether they can better differentiate between recessive and dominant variants. The RNU4-2 locus is very small, and the size of the HDR library used is modest (539 variants), which makes such an experiment manageable, and would substantially increase both the novelty and clinical utility of the results. In my opinion, this experiment would significantly advance the field and make this paper highly novel, fitting for publication in Nature.

Genetic effects that prove to be dominant at the organismal level are often not dominant at the cellular level. It is, therefore, highly challenging to establish a new assay that experimentally distinguishes between dominant and recessive variants, as is especially the case when the mechanisms driving each inheritance pattern are not completely understood. However, agreeing that SGE in diploid cells could, in theory, prove highly informative, we repeated the SGE experiment in triplicate using diploid HAP1 cells. We chose diploid HAP1 because variability in *RNU4-2* and *RNU4-1* expression across cell lines make it difficult to say whether *RNU4-2* variants will lead to phenotypes in other cell lines. Performing the same SGE protocol in diploid HAP1 allows us to directly ask whether known ReNU syndrome variants act dominantly in our model, a question addressable within the timeline of peer review.

These data are shown in **Sup. Fig. 7**. We mostly observe attenuated effects for previously depleted variants, consistent with partial rescue of the cellular impact provided by having a second allele expressed in each cell. However, there is a key difference between the two datasets: in the diploid line, there is no observable impact of ReNU syndrome variants in the Stem III region, whereas all ReNU syndrome variants were significantly depleted in the haploid line. This suggests a different mechanism underlying the T-loop and Stem III ReNU variant effects in HAP1, which may explain the differences in phenotypic severity that have been reported (Nava *et al*. Nature Genetics 2025). This intriguing new result will need further investigation in large studies of ReNU patients.

We have included these new data in the results:
"In an attempt to distinguish recessive and dominant variants experimentally, we performed SGE of *RNU4-2* once more, this time using a diploid population of HAP1 cells selected via fluorescence-activated cell sorting (FACS; see **Methods**). This experiment revealed function scores to be attenuated across the gene due to the presence of the second allele (**Sup. Fig. 7A, B, Sup. Table 1**). Intriguingly, though, all variants assayed in the Stem III region scored neutrally in diploid HAP1, suggesting pathogenic Stem III variants likely impact cell fitness in a manner that is distinct from pathogenic variants elsewhere. For all other regions, function scores between haploid and diploid models were highly correlated (**Sup. Fig. 7C**), indicating fitness effects in diploid HAP1 cells do not delineate dominant and recessive variants *in vivo*." and also in the discussion: "We also performed SGE in diploid HAP1 cells. While function scores from these experiments revealed differences between T-loop and Stem III variants, they were once more unable to distinguish dominant and recessive variants *in vivo*." and have added the relevant methods.

While diploid SGE has succeeded in distinguishing variants that are no longer functionally impactful in the presence of a second allele in the HAP1 system, the assay's inability to discriminate variants that act as recessive or dominant at the organismal level highlight the

challenges of developing such an assay. Indeed, it is highly plausible that the splicing alterations downstream of *RNU4-2* mutation which lead to HAP1 growth phenotypes are not the same as those that cause ReNU syndrome. Importantly to the reviewer's point, the new insights from our patient RNA-seq analyses suggest a clear path forward for distinguishing recessive and dominant variants clinically, as discussed at length in the **Appendix**.

4. For clinicians to use these data in diagnostic variant classification they need to be calibrated such that a strength of evidence can be applied within the ACMG framework. While I appreciate that this framework may change in the near future, it is important to calibrate the variants for use now. I would therefore suggest that the authors follow the recommendations in Brnich et al. 2020 (Genome Medicine) to calibrate these data for clinical use.

Thank you for this suggestion. We have now included a calibration of these data for ReNU syndrome using reported pathogenic and likely pathogenic variants from Nava *et al*. (Nature Genetics 2025) and assayed variants with a combined allele count in UK Biobank and All of Us > 100 as truth sets of pathogenic and benign variants, respectively. This is now explained in Results, with a Supplementary Figure added: "We next used our function scores to assign evidence strengths for clinical variant classification[8]. We deemed the 17 pathogenic / likely pathogenic variants reported by Nava *et al*. and assayed here to be associated with ReNU syndrome and 45 variants with combined allele counts across the UK Biobank and All of Us over 100 to be neutral. A Gaussian mixture model was then applied to determine the odds of pathogenicity (OddsPath) for each variant (see **Methods; Sup. Fig. 5**; **Sup. Table 1**). Within the CR, 69 of 127 (54.3%) variants receive PS3 strong evidence of pathogenicity, including 16 of 18 variants reported to be pathogenic, with the other two variants receiving PS3 moderate or indeterminate evidence. A further 38 (29.9%) variants receive BS3 strong evidence of benignity. As no variants outside the CR have been associated with ReNU syndrome, we refrain from assigning evidence strengths to variants outside the CR."

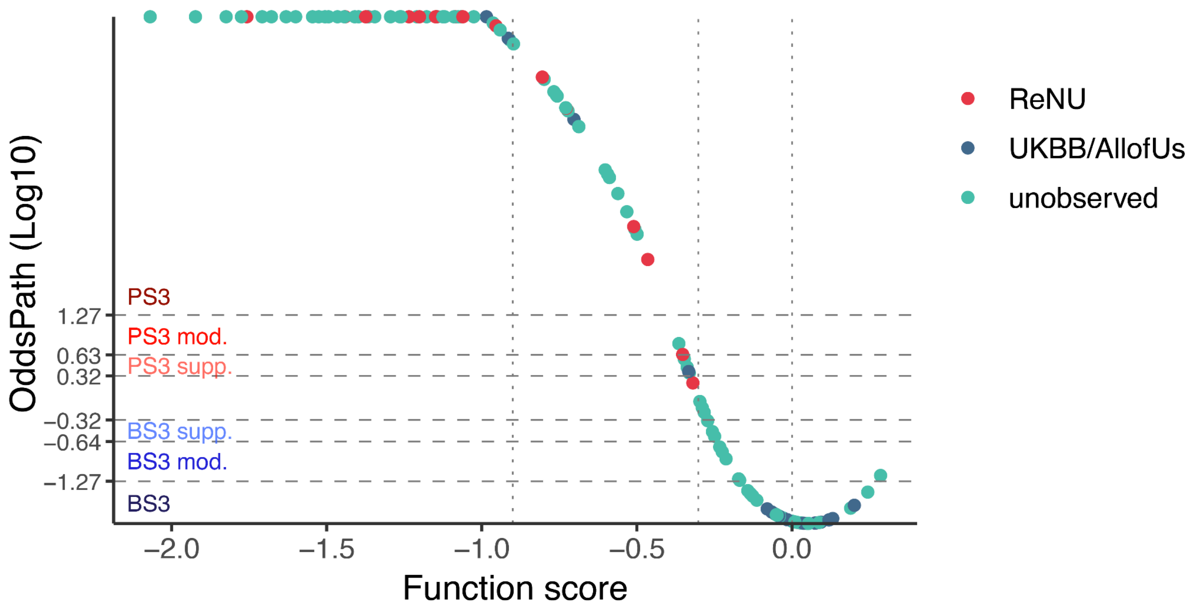

Supplementary Figure 5. Calibration of function scores to evidence for clinical classification of variants in relation to ReNU syndrome. Gaussian mixture modelling was used to estimate odds of pathogenicity (OddsPath). Function scores are plotted against OddsPath values for *n* = 127 variants

within the ReNU syndrome critical region. Vertical dotted lines mark the median of insertion controls (x = 0), as well as thresholds for “moderate” (-0.302) and “strong” (-0.90) depletion. Horizontal dashed lines indicate OddsPath thresholds for assigning evidence strengths in accordance with ACMG guidelines[8]. OddsPath values are capped for variants with function scores below -1.0 to display all points.

It’s worth noting that we have not included a similar calibration for the newly identified recessive disorder. This process would be circular, given our function scores were used to define variants underlying recessive disease, and such circularity is explicitly discouraged. It will, however, be important to perform such a calibration in the future once more independently identified variants are confirmed.

5. The Chan et al 2024 paper included RNA sequencing of patients with ReNU syndrome. It would be very useful if the authors could carry out RNA sequencing of patients with the recessive syndrome associated with RNU4-2 and compare this with the ReNU syndrome data. This may identify clinically useful biomarkers that will facilitate further resolution of VUS.

We now have access to RNA-sequencing data from six individuals with biallelic *RNU4-2* variants, three each from two different centres. As detailed in the summary section above, we have included these data in the companion manuscript. Our rationale for this is that these data fit better alongside the characterisation of the recessive syndrome, and its comparison to ReNU syndrome, both of which are in the companion paper. Excitingly, these data do indeed include a clinically useful biomarker, as individuals with the recessive NDD (but not ReNU Syndrome) have dramatically reduced *RNU4-2* levels and a corresponding increase in *RNU4-1* expression. Hence, the ratio of *RNU4-2* to *RNU4-1* expression could be used as a biomarker for the recessive NDD. These data are described fully in the companion manuscript and in the **Appendix** at the end of this document.

In the current manuscript, we reference these RNA-seq analyses in the discussion: “In our companion manuscript[22], we show through analysis of blood RNA-sequencing data that individuals with biallelic *RNU4-2* variants do not have the ReNU signature of disrupted 5’ splice site selection. Further, biallelic individuals have dramatically decreased *RNU4-2* expression, which is not observed in individuals with ReNU syndrome, supporting a distinct loss-of-function molecular mechanism. As variants in the equivalent regions and nucleotides of *RNU4ATAC* that cause recessive RNU4atac-opathies have been shown to lead to intron retention[30,31], a similar mechanism may underlie recessive *RNU4-2* NDD. However, this was not readily evident in RNA-sequencing analysis in blood[22].”

Minor Comments:
Introduction
1. The authors state that they “developed a strategy” to combat the high sequence homology of RNU4-2 with its homologs and pseudogenes. This is strong phrasing for a minor modification of an established approach – perhaps “identified a method” or “implemented an approach” would be more appropriate.

This has now been edited to the suggested “implemented an approach”.

Methods:
1. Please present details of experimental QC of the HDR library using a high-throughput sequencing method to assess even representation of all designed variants, detect synthesis bias or missing/underrepresented variants, and quantify the proportion of wildtype and PAM-only sequences.

We previously reported: "All variants were observed in the library and day 4 at a frequency higher than 10^{-4}, and were therefore included in downstream analyses." With the addition of the third replicate, this is still correct. Further, the requested QC details are now presented in a new **Sup. Fig. 1**.

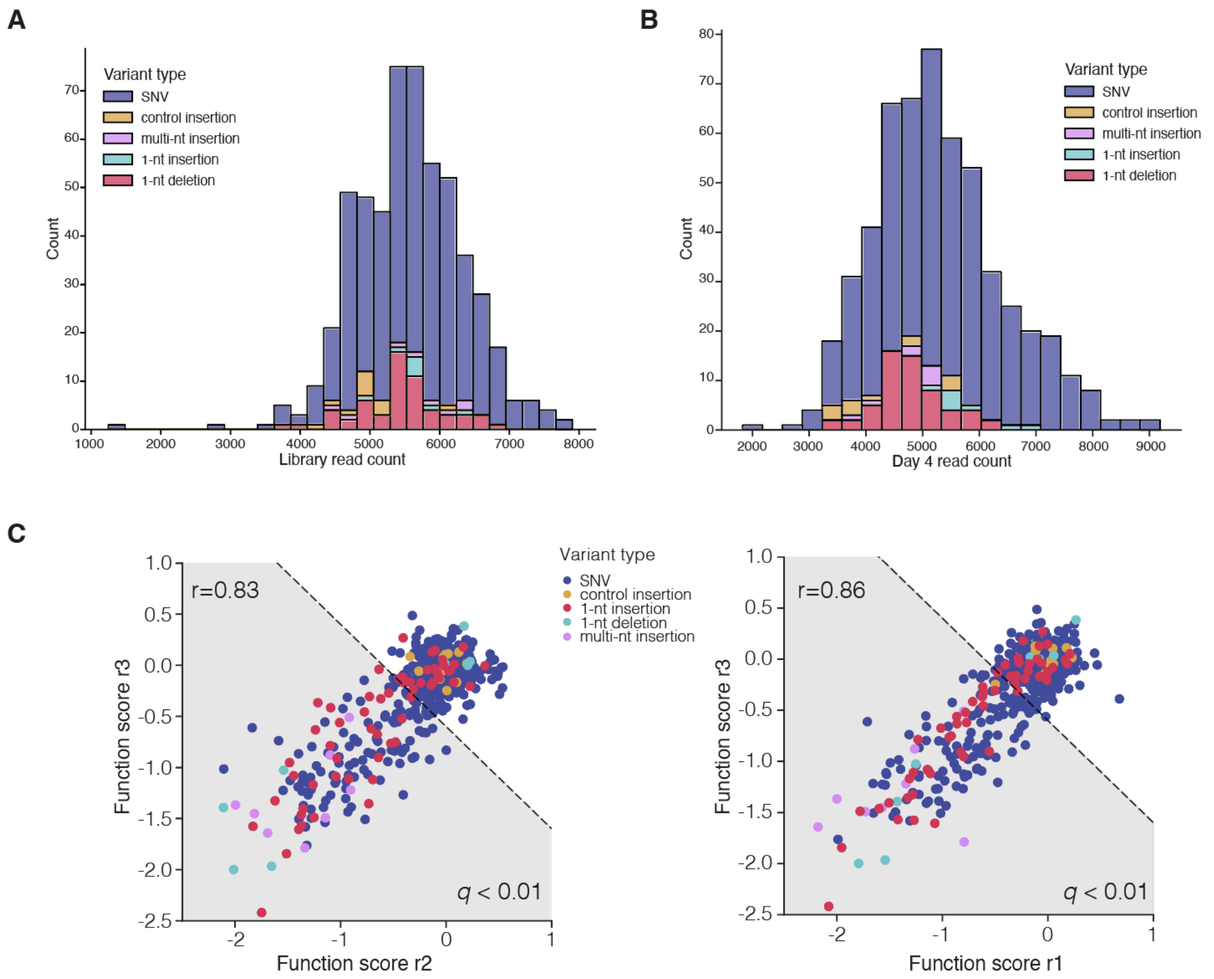

Supplementary Figure 1. Quality control metrics for *RNU4-2* SGE experiments. A. The distribution of variant read counts in the HDR library is plotted for all n = 539 variants included in library design. Of reads from the HDR library, 0.0068% and 4.6% matched unedited reference and PAM-edit only, respectively. **B.** The distribution of variant read counts in day 4 gDNA is plotted, with counts averaged across biological replicates. **C.** Inter-replicate function score correlations are plotted, with Pearson's r shown and variants coloured by mutation type.

2. The authors state that all but one variant known to cause NDD were included in the assay (line 110), but do not state the reason for this exclusion, or why some VUS were not tested (presumably they were reported after the HDR library was designed?).

You are correct. We designed the assay before the Nava *et al*. 2025 paper was in preprint. At this point, only single base insertions and SNVs had been reported. The n.72_73del two base deletion variant reported by Nava *et al*. was not captured by our assay design. This is already mentioned in our discussion. To make clear the reason for this, we have expanded the relevant Results sentence to state "... including all but one variant known to cause NDD (omitting n.72_73del which was reported after assay design; **Fig. 1A**)".

3. Include a more detailed description of how the PAM-disrupting mutation was selected (e.g. what is the corresponding CADD/PhyloP score of the position of this mutation?).

We have revised the Methods section as follows: "A PAM-blocking mutation was introduced 27 nt upstream of the coding sequence (chr12:120,291,930-C-G) via primer overhang extension during PCR. The location of the PAM-disrupting edit was selected to minimise re-cutting by Cas9, converting a 5'-GGG PAM sequence to 5'-GCG. The PAM-disrupting edit had a CADD score of 4.20 (Phred) and a 100-vertebrates PhyloP score of 0.11."

4. Please clarify what the allele count threshold used to define 'population negative control variants' is, and why it was chosen.

For the majority of our analyses we did not include any threshold on the population data, annotating variants solely based on presence or absence in population controls, defined to be participants in UK Biobank or All Of Us. We now make this more clear in the legend of **Fig. 2**, "The gray dashed line separates variants absent from UK Biobank and All of Us (combined AC = 0) from those observed in at least one dataset (combined AC > 0)." For the abovementioned calibration for ACMG/AMP evidence strength, however, we used an allele count >100 across the UK Biobank and All of Us cohorts combined. This is described in the methods.

Results:
1. 539 variants is a modestly size SGE library. Given the mutational rate in this region, this is somewhat of a missed opportunity to have not tested indel insertion along the length of the RNU4-2 transcript.

This was a relatively difficult SGE experiment to do, due to there being very limited gRNA design options that uniquely target *RNU4-2* and not homologous sequences elsewhere in the genome. Indeed, we piloted transfection of multiple gRNAs before proceeding with one that did not cause excessive cell death. Therefore, while the number of variants was 'modest', the assay required a high number of input cells to obtain clean results. Obviously, in hindsight, we would have liked to include more variants, but at the time of assay design, disease-causing variants had only been observed in the central 18-nt ReNU region, so we concentrated our efforts there and prioritised obtaining clean data.

2. Define the "six distinct regions where variants score as depleted" mentioned (line 125) in genomic coordinates.

Thank you for highlighting the need to be clear when we are mentioning depleted regions as these are relevant to clinical interpretation. With that, we have revised the text so that we do not mention these regions until we discuss them in the context of the recessive NDD towards the end of the manuscript. Where we do then mention these regions, in the penultimate paragraph of the results section, we include the nucleotide positions. In **Fig. 1**, and the associated text, we focus on the known structural elements of U4 to better align with the text in these initial sections.

3. It would be useful to compare the SGE data to in-silico variant effect prediction tools other than CADD. For example, are there popEVE scores available for RNU4-2?

popEVE, like the vast majority of *in silico* tools usually compared against SGE scores, does not work for non-coding RNAs, scoring only variants in protein-coding regions of the genome. We are very limited in tools we can assess against which work genome-wide. We chose CADD as the only widely-used genome-wide tool.

To supplement our CADD comparison, we have now explored to what extent computationally predicted impacts on RNA structure correlate with function scores, using ΔΔG predictions for minimum free energy of the U4:U6 complex from ViennaRNA. The results of this analysis are now included in the results: "We also observe only a weak correlation of SGE function scores with changes to U4/U6 RNA binding stability predicted by ViennaRNA (ρ = -0.27, $P = 4.5 \times 10^{-10}$; **Sup. Fig. 3A**). The observed effect is limited to specific regions, most notably Stem II (ρ = -0.79, $P = 5.0 \times 10^{-10}$). In contrast, no significant correlation is observed in the T-loop or Stem III and overall, ΔΔG values from ViennaRNA do not classify ReNU syndrome variants as well as SGE (ROC-AUC 0.72 vs. 0.93, respectively; **Sup. Fig. 3B**)." and in a new **Sup. Fig. 3**. Given the relatively poor performance of the RNA structure predictions we caution against using RNA structure predictions to inform clinical variant classification at this stage.

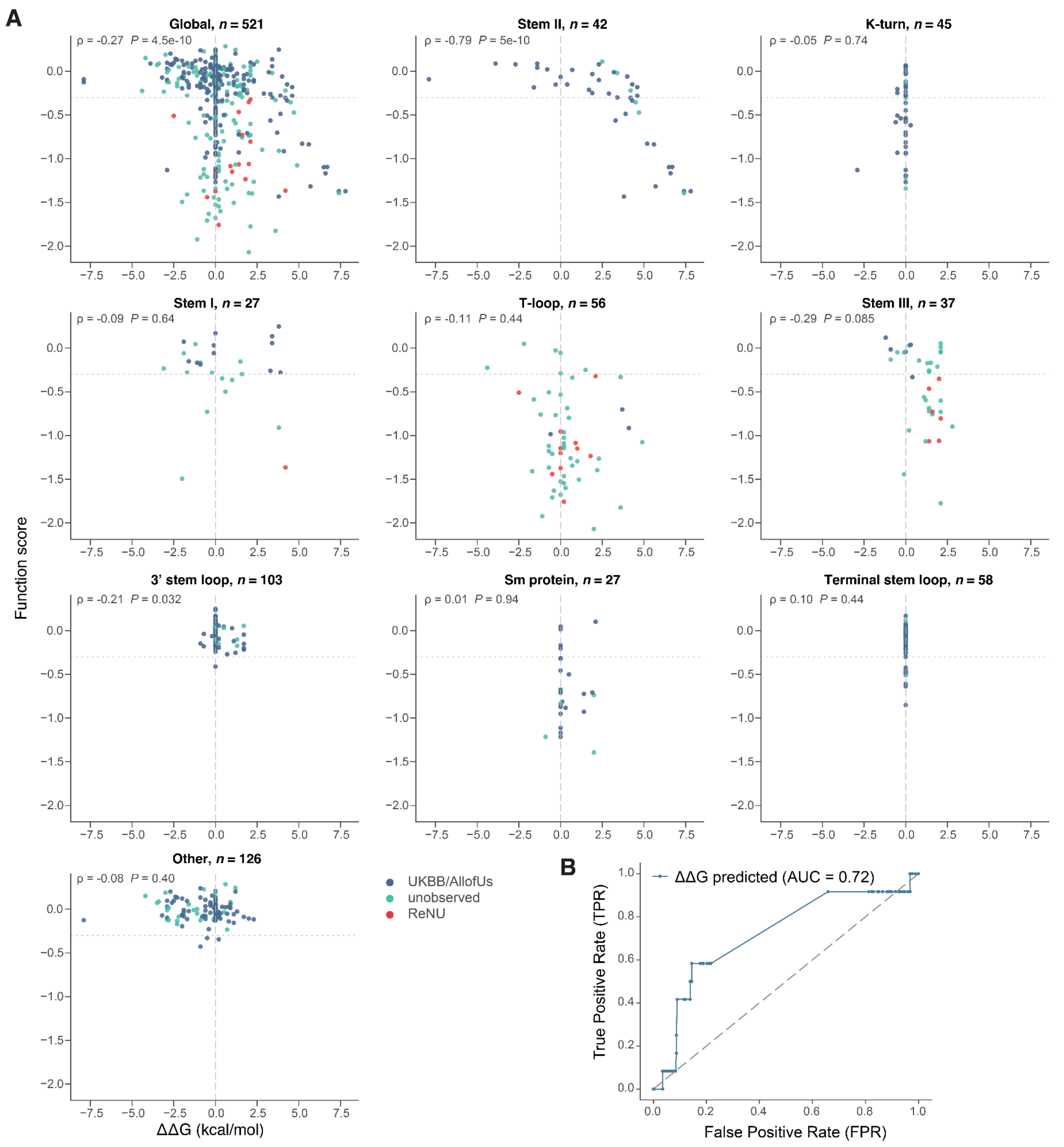

Supplementary Figure 3. Correlations between function scores and predicted effects on RNA binding stability. ViennaRNA was used to predict the effects of variants (n = 521) on the minimum free energy of U4/U6 RNA binding compared to reference (ΔΔG). **A.** Predicted ΔΔG values are plotted versus function scores for the whole transcript, as well as for individual regions (Spearman's ρ). **B.** ROC curve for classifying ReNU syndrome variants from population controls using ViennaRNA-predicted ΔΔG values (AUC=0.72).

4. How does the projected prevalence of the recessive NDD associated with RNU4-2 compare to that of ReNU syndrome? This information is in the accompanying clinical manuscript but should be included in the SGE paper also.

This is very hard to define at this stage, especially given the increased prevalence of recessive disorders in consanguineous populations and the bias in individuals who have access to genome sequencing (which is needed to identify variants in *RNU4-2*). While we do

not feel comfortable estimating the prevalence at the moment, we have added the following to the discussion of the manuscript, with reference to the companion manuscript for more details: "While we cannot yet determine the prevalence of the recessive NDD, SGE-depleted variants outside of the ReNU CR are found in 0.12% and 0.094% of individuals in the UK Biobank and All of Us cohorts, respectively. Hence, the recessive NDD is rarer than ReNU syndrome, but the prevalence is likely increased in populations with higher rates of consanguinity[22]."

5. Figure 1C/D. It is not immediately apparent that the figure legend for Figure 1D also applies to 1D. Either put C and D on the same row and keep the one figure legend. Alternatively, add a comment in the figure legend that the legend for Figure1C is the same as that for Figure 1D.

Thank you for pointing out that this wasn't clear. We have now included the following text at the end of the legend to note this: "Points in **C** and **D** are coloured by variant type with a single legend included for these two panels."

6. Figure 1D. It would be useful to highlight visually in the figure the "six distinct regions where variants scored as depleted" mentioned in the results before the authors reference Figure1D. For example, it is unclear where the (assumed) right-most region finishes when interpreting this by eye. Clinically it is important to know what the boundaries of these regions are in genomic coordinates.

As mentioned in the response to point two above, we have now removed mention of these 'regions' early in the manuscript, as the context of the recessive disease is important for their interpretation. In the final section of the results, we reference the regions alongside their corresponding nucleotide positions. In **Fig. 1D**, we believe showing the known structural regions of U4 is more relevant, and we have updated the figure annotations to provide more granularity with regards to the 3' stem loop regions.

7. Figure 1A/C/D. The purple colour used for multi-nt insertions is hard to see. Could a more contrasting colour be chosen?

We have altered the colouring in **Fig. 1** to include a greater contrast and agree that this is now clearer.

8. Figure 2D. It's confusing that there are two variants coloured in blue, which have an AC=0 in UKBB. Presumably, these are variants that were seen in the All of Us cohort. Would it be possible to plot instead the sum of the allele counts in both cohorts against the SGE score?

Thank you for making us aware that this was confusing as presented. We have now altered **Fig. 2D** to show the sum of the allele counts across UK Biobank and All of Us, as suggested.

9. Figure 3b. It would be useful to give the number of patients phenotyped for each category above the plot (ie document n=x above the labels "ID, "GDD", "speech" and "seizures". This will be a useful guide to clinicians if they see a patient who does not fit this pattern.

We have now added this information to **Fig. 3B**. In addition, these counts are within **Sup. Table 2** and we have now referenced this fact in the legend so that readers can easily access this data. Specifically this now reads: "Sample sizes for each phenotype are shown, and full data, including statistics for comparisons between groups, are included in **Sup. Table 2**."

10. Figure 4. Shaded teal regions are a very similar colour to that of neutral SNVs. Could a more contrasting colour be used?

We have now adjusted the colour of these regions to add greater contrast.

11. Figure 4. "Black" triangles appear grey. Please change the colour or the legend description for clarity.

Thank you for spotting this discrepancy. The figure legend should have read "grey triangles" and has now been corrected.

12. Supplementary Figure 1: it is not clear to my why just 12 ReNu syndrome variants were included, why not all known dominant variants?

As SNVs were the only variants introduced throughout the whole gene, we focused this gene-wide analysis on SNVs to avoid potential confounding from only introducing indels to the critical region. The ROC-AUC calculation in **Fig. 1** was also in comparison to CADD scores, which are typically only pre-computed for SNVs.

Importantly, the overall trend is the same if all 18 ReNU variants are considered. For instance, perfect classification is obtained (AUC = 1.00) when defining controls as variants with UK Biobank allele counts greater than 60 (43 control variants) or All of Us allele counts greater than 40 (52 control variants).

13. Supplementary Figure 1: Differences in dot sizes indicating number of variances are subtle. Contour lines or plot labels showing exact variant counts for key regions might help interpretation.

Thanks for pointing this out. We agree and have updated the figure (now **Sup. Fig. 2**) by removing the dot sizes and instead labelling the number of control variants retained at each of several allele count thresholds.

14. Supplementary Figure 2. Include a description of the vertical red dashed lines.

This annotation has now been added to the figure legend of what is now **Sup. Fig. 4**. Specifically, "The vertical red dashed lines represent the boundaries of the 18 nucleotide ReNU CR reported by Chen *et al.*[1] drawn to include insertions at n.61_62 and n.79_80."

15. Thank you for providing all of the data as a clearly laid-out supplementary file. Please could you include reference transcript ID within the HGVS ID given?

The reference transcript ID has now been added to the HGVS as requested.

Discussion:
1. Where the authors list “ascertainment bias, germline selection, and/or an elevated mutation rate” (line 48) as possible explanations for the high recurrence of the single nucleotide insertion (n.64_65insT) observed in individuals with ReNU syndrome, the discussion only addresses ascertainment bias. I think it would be appropriate to briefly reiterate and cite the discussion of these hypotheses made in the Chen, Y. et al. 2024 in the discussion of this paper, or explain why ascertainment bias is thought to be the most likely explanation.

We do not believe that ascertainment bias based on phenotypic severity is the most likely explanation, but it is the only one of the possible explanations for which we have any insights from these SGE data. It did not seem appropriate to discuss the alternatives in detail when they are not supported or refuted by the data presented. Nevertheless, we have expanded this text to include mention of the alternative hypotheses. It now reads: “This result could argue against high recurrence being the result of a particularly damaging functional effect driving ascertainment, suggesting that positive selection in the female germline or an elevated local mutation rate might be more likely explanations. However, we cannot rule out the possibility that this variant leads to unique changes in splicing not reflected in SGE function scores.”

References:
Appropriate papers are cited throughout to give an overview of the existing state of the field. However, Greene et al. 2024 reported that the two distinct regions were associated with ReNU syndrome causing variants: (n.62–70 and n.73–79). Although the newly defined 4-nt region (n.75-78) does not exactly match that of Greene et al. 2024, the 9-nt region (n.62-70) was correctly identified by Greene et al, and it would seem reasonable to acknowledge this.

We had not initially included this comparison as all papers to date on ReNU syndrome have reported that ReNU variants map to the two structures: Stem III and the T-loop. Hence, the two region definition is not unique to the Greene *et al*. paper. Nevertheless, we have now included a direct comparison to the regions mentioned in the Greene et al. paper. Specifically, we state: “While the T-loop region matches that reported by Greene et al.[2], the critical region overlapping Stem III is 3-nt smaller than previously suggested.”

Reviewer #3

- The SGE function score is solely based on a cell growth readout. Yet, given the key role of U4 snRNA in splicing, how the function score relates to splicing should at least be investigated for a subset of variants (e.g. selected ReNU vs. UKB/AllofUs vs. unobserved variants in different regions, variants with strong vs. moderate function scores). For example, selected variants could be introduced in cells followed by RNA-seq and/or using a targeted method to assess splicing events previously linked with ReNU. Pooling variants that were shown to have normal, moderate or strong function scores, and comparing splicing profiles between these pools could also be done. Such experimental validation would significantly strengthen the impact of the study and validate the use of a cell growth readout for assessing the pathogenicity of snRNA variants. Alternatively, the authors previously reported (Nava et al. Nat Genet 2025) that ReNU patients show altered 5’SS usage in lymphoblasts

and that splicing profiles can discriminate between mild and severe cases. The authors could at least show whether “strong” and “moderate” function scores correlate with the extent of splicing alterations in those patients.

Thank you very much for this suggestion. We have now included a PCA similar to that included in Nava *et al*. (their Fig. 6A) with the ReNU patients recoloured by the SGE categories of “strong” and “moderate” as **Fig. 3C**. The following text has been added to describe these new results: “To test whether the strength of SGE depletion also correlates with the extent of splicing disruption observed in individuals with ReNU syndrome, we repeated a second analysis from Nava *et al*. We re-generated a principal component analysis (PCA) of percent spliced in (PSI) values for 5’ splice sites that differed significantly in usage between ReNU cases and controls[4]. Individuals with strong and moderate SGE function scores clustered separately, with the strong variant individuals being more distant from controls (**Fig. 3D**).”

In addition, we have further investigated the impact of the variants on splicing using RNA-sequencing data from six patients, and include the results of this in our companion manuscript. These data are shown in the **Appendix**. Specifically for your point, we show that the recessive *RNU4-2* patients do not show the ‘ReNU signature’ of 5’ splice site disruption. We further investigated whether there was any increase in intron retention in these individuals, based on the mechanism underlying variants in the same functional regions of the minor spliceosome U4, *U4ATAC*, but did not observe a signal. This is not overly surprising given that high levels of global intron retention would likely be embryonic lethal, and aligns with similar findings in *RNU2-2*. We now mention these results in the discussion of this current manuscript: “In our companion manuscript[22], we show through analysis of blood RNA-sequencing data that individuals with biallelic *RNU4-2* variants do not have the ReNU signature of disrupted 5’ splice site selection. Further, biallelic individuals have dramatically decreased *RNU4-2* expression, which is not observed in individuals with ReNU syndrome, supporting a distinct loss-of-function molecular mechanism. As variants in the equivalent regions and nucleotides of *RNU4ATAC* that cause recessive RNU4atac-opathies have been shown to lead to intron retention[30,31], a similar mechanism may underlie recessive *RNU4-2* NDD. However, this was not readily evident in RNA-sequencing analysis in blood[22].”

- Related to the previous point, the authors show that neighboring variants within the ReNU critical region differ in their function score, including variants with normal, moderate and strong function scores. Do the function scores correlate with predicted impacts on U4 secondary structure and/or base pairing with U6 snRNA (e.g. using RNA structure prediction software)?

This is a good suggestion, though we are cautious about the use of RNA structure prediction tools given that the structure of U4 snRNA is presumably constrained not only by sequence and through binding to U6, but also by the protein components of the spliceosome. Without a model that can predict structural changes in the context of the full U4/U6.U5 tri-snRNP, we are hesitant to read heavily into the results. Nevertheless, we do now include an analysis of U4/U6 binding energy predicted using ViennaRNA, both across the entire gene, and within each functional region. We have included this analysis in the results: “We also observe only a weak correlation of SGE function scores with changes to U4/U6 RNA binding stability predicted by ViennaRNA (ρ = -0.27, $P = 4.5 \times 10^{-10}$; **Sup. Fig. 3A**). The observed effect is

limited to specific regions, most notably Stem II (ρ = -0.79, *P* = 5.0x10^{-10}). In contrast, no significant correlation is observed in the T-loop or Stem III and overall, ΔΔG values from ViennaRNA do not classify ReNU syndrome variants as well as SGE (ROC-AUC 0.72 vs. 0.93, respectively; **Sup. Fig. 3B**).” and have added the relevant methods.

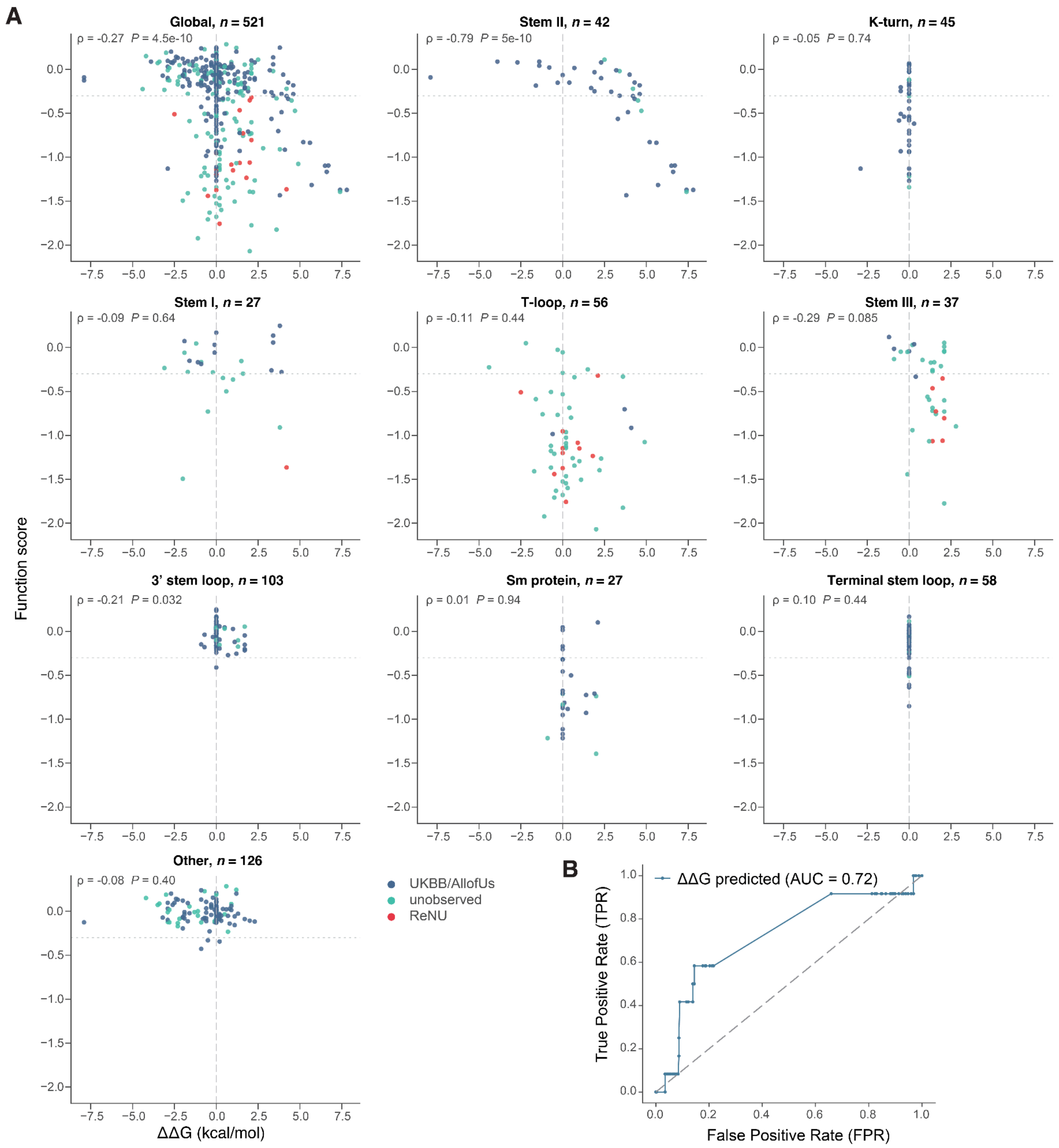

Supplementary Figure 3. Correlations between function scores and predicted effects on RNA binding stability. ViennaRNA was used to predict the effects of variants (*n* = 521) on the minimum free energy of U4/U6 RNA binding compared to reference (ΔΔG). **A.** Predicted ΔΔG values are plotted versus function scores for the whole transcript, as well as for individual regions (Spearman’s ρ). **B.** ROC curve for classifying ReNU syndrome variants from population controls using ViennaRNA-predicted ΔΔG values (AUC=0.72).

- While the overall correlation between replicates is good, some positions show more variability between replicates, including some ReNU variants that are just below the function

score threshold when considering the average between replicates, but with one replicate above threshold. Variability between replicates should be considered when assigning variants to moderate or strong categories.

In response to a request from reviewer #1, we have now performed a third replicate of the entire experiment. This third replicate correlates highly with the other two and gives more confidence in the function score estimate for each variant. Additionally (and as detailed above), we have also calibrated our function scores for variants in the critical region to evidence strengths for clinical classification using Gaussian mixture modelling, in line with established ACMG guidelines. It is worth noting that this independent calibration assigns 'indeterminate' evidence (neither in favour or against pathogenicity) to variants with function scores between -0.28 and -0.32, a range that includes the significance threshold established independently using control insertions. Furthermore, neither PS3-strong or BS3-strong evidence is assigned to any variants scoring between -0.46 and -0.15, reflecting the greater degree of uncertainty for near the threshold. This calibration is now included as **Sup. Fig. 5**.

Given that the threshold chosen to distinguish moderate from strong is already arbitrary (chosen to ensure enough variants/individuals are in each category), and in light of the increased confidence in the point estimate provided by the third replicate, we don't believe that further adjustment is appropriate. It is also worth noting that all variants scoring near the threshold between moderate and strong have highly significant q-values.

- In Fig. 3B, do the trends hold if separated by variants? Are there specific variants that drive this effect?

There are not sufficient numbers of individuals with any variant other than the highly recurrent n.64_65insT variant to perform separate analyses. That said, given that the observed result could be driven by n.64_65insT alone, we repeated this analysis excluding these individuals, however we saw consistent results. We have included mention of this in the results: "These results remained consistent when excluding n.64_65insT from the analysis (i.e. the result is not driven by the recurrent insertion variant alone) and when using a UMAP representation (**Sup. Fig. 6**)." and have added an associated Supplementary Figure.

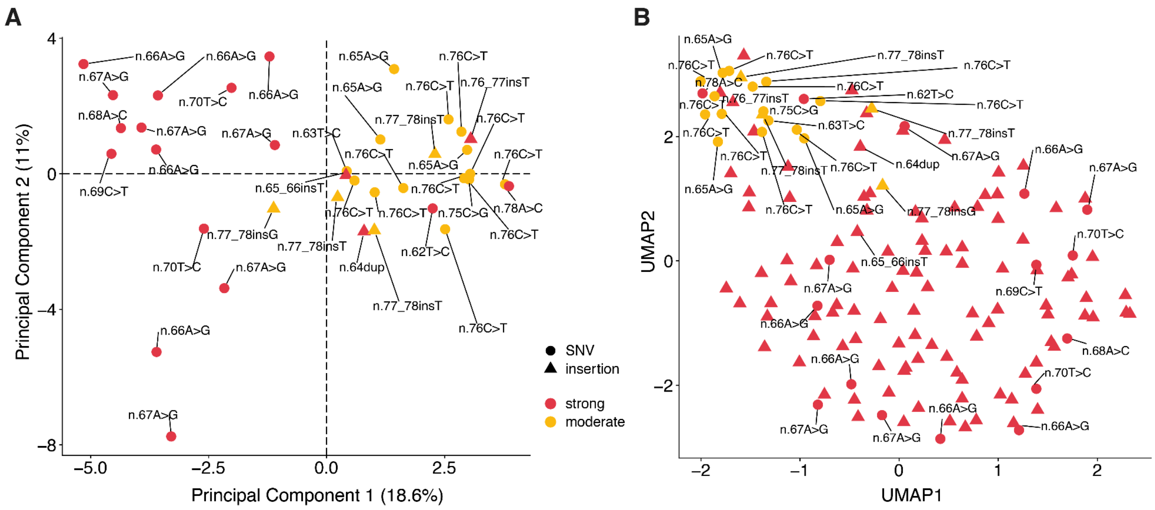

Supplementary Figure 6. Phenotype clustering of ReNU patients. A. PCA clustering as in Figure 3A but removing individuals with the recurrent n.64_65insT variant. **B**. Phenotype clustering of all individuals represented in Figure 3A using a UMAP representation.

Minor comments:
- The use of the HAP1 cell line should be justified in the text when it is first introduced. While its haploid nature facilitates SGE, it is a distinct cellular type from those affected in NDD. Similarly, the authors should justify why using cell growth as a readout for NDD pathogenicity is appropriate.

We fully agree with the reviewer's point. Indeed, there was no expectation HAP1 should necessarily work as a model *a priori*. We thought attempting growth-based SGE in HAP1 was reasonable because HAP1 has worked well for several protein-coding genes despite not resembling the cell type of physiological relevance in any case. Of course, the greatest evidence of its value is the data presented throughout the paper.

To better justify the use of HAP1, we now introduce the cell line in Results as follows: "Lacking established models for assaying *RNU4-2* variants, we chose to perform SGE in HAP1, a haploid human line in which growth effects have accurately distinguished pathogenic variants across several protein-coding genes[10,12,14,16–18]."

- Fig. 3B: what the error bars represent should be specified (between variants?). A binomial test with default features (p=0.5) doesn't seem appropriate to obtain 95% confidence intervals, given that there are three phenotypic categories for each function score category.

Fig. 3B shows only raw proportions of individuals within each group. The error bars represent the 95% confidence interval on the proportion, given the sample size. This is specified in the figure legend. There is no data from any statistical test plotted here, and therefore a multiple testing correction was not applied. Data from statistical tests between the groups are included in Supplementary Table 2, with the appropriate details of the statistical test and multiple testing correction included there. We have added the following sentence to the figure legend to make this clear: "Sample sizes for each phenotype are shown, and full data, including statistics for comparisons between groups, are included in **Sup. Table 2**."

Referee #3 (Remarks on code availability):
I have reviewed the secondary code and was able to run it with minor modifications (e.g. changing the name of the file RNU4_2_ST1_15042025.csv and skipping the header line). In the Python code, additional comments to explain each filter would be useful.

Thank you very much for testing our code and for making these suggestions to increase readability and utility. We have now updated the code to include more comments on the filters and have made alterations to enable the file to be read in without additional modifications.

Reviewer #4

Major points:
1. ReNU is not a new disease entity nor is SGE a new technique. Thus, the major novelty of the paper is twofold: (1) a repertoire of variants and predicted pathogenicity, and (2) a hitherto unreported recessive NDD caused by RNU4-2. The latter left me wanting much more in terms of answering the question of why phenotypes are clinically distinct from ReNU. Neither manuscript begins to answer this question sufficiently or speculates to satisfaction. RNAseq should be performed on cell lines from affected individuals with the recessive RNU4-2 NDD for comparison with the ReNU samples reported by Chen et al. Even if access to samples from affected individuals is not possible, the authors have demonstrated their expertise with CRISPR/Cas9 and could edit these regions of the gene to simulate biallelic variants so that global impacts on splicing can be determined. This key experiment would offer a first glimpse of the molecular differences between the two RNU4-2 disorders, which is much needed to meet the bar for publication in Nature.

We now have access to RNA-sequencing data from six individuals with biallelic *RNU4-2* variants, three each from two different centres. As detailed above, we have included these data in the companion manuscript (also detailed in the **Appendix** at the end of this document). Our rationale for this is that these data fit better alongside the characterisation of the recessive syndrome, and its comparison to ReNU syndrome, both of which are in the companion paper. These data beautifully show the difference in mechanisms underlying variants in the different regions, with the biallelic variants leading to a loss of *RNU4-2* expression which is not observed in individuals with ReNU Syndrome.

In this current manuscript, we have now also included data to support that the SGE scores correlate with the level of disruption to splicing in ReNU syndrome (**Fig. 3C**). We also include reference to the RNA-sequencing results in the companion manuscript in the discussion: "In our companion manuscript[22], we show through analysis of blood RNA-sequencing data that individuals with biallelic *RNU4-2* variants do not have the ReNU signature of disrupted 5' splice site selection. Further, biallelic individuals have dramatically decreased *RNU4-2* expression, which is not observed in individuals with ReNU syndrome, supporting a distinct loss-of-function molecular mechanism. As variants in the equivalent regions and nucleotides of *RNU4ATAC* that cause recessive RNU4atac-opathies have been shown to lead to intron retention[30,31], a similar mechanism may underlie recessive *RNU4-2* NDD. However, this was not readily evident in RNA-sequencing analysis in blood[22]."

Minor points
1. The authors mention the challenges to specifically target RNU4-2 due to high sequence homology with RNU4-1. Did they confirm specificity by sequencing RNU4-1 in the HAP-1 cells used for SGE? A brief mention in the main text would offer reassurance.

We picked a 23-bp target (protospacer plus PAM) that differed from the homologous sequence upstream of *RNU4-1* by eight nucleotides, including a mismatch in the PAM sequence, such that the chance of editing *RNU4-1* was minimal. Indeed, we sequenced the *RNU4-1* locus from edited cells and did not observe any variants consistent with editing. We now confirm this in Results: "Editing was confirmed by sequencing to be specifically targeted to *RNU4-2*, and not *RNU4-1*." We also add clarity regarding design to Methods:

“The selected gRNA was not predicted to target *RNU4-1*, owing to eight mismatches occurring in the protospacer and PAM.”

2. Although the eight multinucleotide insertions in the CR that were included as positive controls were all depleted (functional scores well below the -0.39 threshold), it is intriguing that not all the 1-nt insertions were scored as pathogenic (Fig 1D, especially in Stem III). Can the authors explain why this might be?

There are only three single base insertions within the two redefined critical regions that are not significantly depleted in the assay. Two of these (n.61dup and n.61_62insG) are very close to the threshold of significance (-0.297 and -0.283, respectively) and are very close to the boundary of the critical region in the T-loop - all tested variants at the adjacent position (n.61) score as neutral. Therefore, the scores of these two non-significant insertions likely represent a milder impact on *RNU4-2* function. The nuance around function scores near the threshold of significance is now better reflected in our revised discussion (see point 4 below).

The other (n.76dup) is within Stem III and has a function score of -0.015; this variant is one of only three insertions within the two redefined critical regions that are observed in population controls, supporting its neutral score. Notably, as nts 74 to 76 are all cytosines, this particular insertion preserves the intact Stem III sequence.

3. Can the authors please confirm that in the individuals with recessive RNU4-2 NDD that there were no other candidate causal genes or variants elsewhere in the genome; particularly any other spliceosome related ncRNA or CNVs. This may be more appropriate to include in the companion paper but deserves a mention.

Thank you for this important point. We have included more details about prior analysis in the companion paper, but have now also included here: “None of the individuals had an existing genetic diagnosis that fully explained their observed phenotypes (see **Methods**).” in the relevant results section. We have also added the following sentence to the methods: “All individuals had prior genome analysis including investigation of variants in known NDD genes and large structural variants. One individual (individual 17) had a reported likely pathogenic variant in *GLI3*, however, this variant did not explain all of their reported phenotypes (see companion manuscript for more details[22]).”

4. The discussion paragraph beginning on line 347 states “we cannot exclude the possibility that variants with more subtle effects may be clinically relevant”. It would make sense to also state the following (or something similar) “nor can we exclude the possibility that variants that score at or near the -0.39 function score threshold are benign, potentially representing false positives that should be interpreted with caution, particularly when clinical decisions are at stake”

We agree that there is also some uncertainty with variants that just pass the significance threshold, however, given how this threshold was defined ($q < 0.01$), it is rather stringent. Regardless, we have now included this caveat, as suggested. Further, in response to a suggestion from reviewer #1, we have also now calibrated the SGE scores for use within the ACMG/AMP framework. We have worked this into an expansion of the discussion with the following text: “While all variants associated with ReNU syndrome scored below this

threshold, we cannot exclude the possibility that variants with more subtle effects may be clinically relevant, particularly in relation to recessive disease. Conversely, we can't fully exclude the possibility that variants that score just below the -0.302 function score threshold are benign and represent false positives. The calibration of function scores to evidence strength for ReNU variant classification reflects this, as variants were not assigned PS3 strong evidence in favour of pathogenicity unless their function scores were below -0.45."

5. Supplementary Table 1 would benefit from a column that has the calculated mean function score. Although any reader could do this themselves, it would make it easier to cross-reference specific variants highlighted in the main text and figures.

Thank you for pointing out the lack of clarity on this. Supplementary Table 1 did include mean function scores under the heading "function_score". We acknowledge this is confusing, and now have labelled this column "function_score _mean" and have put these values in bold to help distinguish them.

6. The Figure 4 legend mentions black triangles corresponding to homologous positions of RNU4ATAC. These triangles appear to be gray in the figure; please modify for consistency.

Thank you for spotting this discrepancy. The figure legend should have read "grey triangles" and has now been corrected.

7. A minor cosmetic point, but the horizontal dashed line in Figure 2A is barely visible. Changing the shade or color would assist readers.

Thank you for pointing out that this is not clear. We have altered the shade of this, as well as similar lines in panels B and D, to make them more evident.

Response to Reviewers for "Saturation editing of RNU4-2 reveals distinct dominant and recessive disorders" by De Jonghe *et al.*

We thank the reviewers for their positive feedback on the revised manuscript. We address their remaining concerns in blue as follows.

Referee #1

…We appreciate the authors caution in not calibrating the assay for variants outside of the ReNU CR. However, in practice, clinicians will use these data to assess variants in this region, and will look to these two publications for guidance in how to use these data in clinical variant classification. How do the authors suggest the SGE data be used in regions outside of the ReNU CR? we presume the authors recommend ascribing 'Supporting evidence' if a variant has a statistically significant SGE score (pending calibration of SGE scores using an independently-ascertained cohort)? We think describing this clearly in the discussion is important (in both manuscripts).

We've have edited the Discussion as the reviewer has suggested:

> "While we showed that function scores for variants within the ReNU CR can provide strong evidence for clinical interpretation, we were unable to calibrate our assay for variants outside the ReNU CR due to a lack of independently defined pathogenic variants in these regions[8], as all individuals with recessive NDD were identified on the basis of function score. While we anticipate that our SGE data will prove highly useful for delineating variant pathogenicity for recessive disease, until orthogonal calibration can be performed, we recommend PS3 supporting evidence be assigned to significantly depleted variants outside the CR."

It is not currently sufficiently clear in this manuscript that not all individuals with biallelic RNU4-2 variants have statistically significant SGE results. For example: the following additions would be helpful:

We agree this is a key point to highlight. Among the 18 additional individuals with biallelic variants that were not *both* SGE-depleted, 3 individuals were found with two non-depleted variants. Most (11) had one SGE-depleted variant, though 4 had a variant not scored in the assay but predicted to be deleterious based on scores of variants nearby. We have therefore included the reviewer's suggested sentences with modifications for accuracy:

On page 11: "The clinical phenotypes of the twenty identified NDD individuals are characterised as part of a broader cohort (total n = 38) in a companion manuscript. The

18 additional individuals reported in this broader cohort have biallelic RNU4-2 variants with non-significant functional scores".

This suggestion has been revised and added to the manuscript:

> "The clinical phenotypes of the twenty identified NDD individuals are characterised as part of a broader cohort (total *n* = 38) in a companion manuscript[21]. The eighteen additional individuals reported in this broader cohort all have biallelic *RNU4-2* variants, but at least one variant had a non-significant function score or was not scored with SGE."

On page 12: "This NDD is described comprehensively in a companion manuscript, in which we also expand the cohort to include 38 individuals: the 20 individuals with biallelic variants with significant function scores that are presented here, and 18 additional individuals with variants that are not significantly SGE-depleted, but are found in the same functional regions of RNU4-2."

This suggestion has been revised and added to the manuscript:

> "This NDD is described comprehensively in a companion manuscript[21], in which we also expand the cohort to include 38 individuals with biallelic *RNU4-2* variants: the 20 individuals presented here with significant function scores for both variants, and 18 additional individuals harbouring variants in the same functional regions with at least one variant that was not significantly depleted or not assayed by SGE."

Referee #3

Fig. 6A of Nava et al. reports 19 patients and 21 controls, whereas Fig. 3B of this manuscript used 19 patients and 20 controls, resulting in slight changes to the PCA. While the results may not change substantially, it would be more transparent to indicate why one control was removed, which apparently changes the number of significant A5SS events from 111 to 101.

One control individual was removed because this person was found to have biallelic *RNU4-2* variants and is now classified as a recessive case. We have edited the Methods to make this explicit:

> "rMATS-turbo (v4.3.0)[35] was run on 19 ReNU samples and 20 controls (excluding one individual previously deemed a control by Nava *et al.* who was here found to be a recessive *RNU4-2* case)"

For the RNU4-1 and RNU4-2 expression analysis in the companion paper (but mentioned in the Discussion of this manuscript), the two transcripts have >97% sequence identity. Are the 4 mistmaches sufficient for the majority of reads to be uniquely mapped to one of the two transcripts in the DRAGEN pipeline? Otherwise, how were reads mapping to both transcripts handled?

We have looked extensively for mismapping between *RNU4-1* and *RNU4-2* in various contexts, both computationally, and through manual review of read data on IGV. We do not see any evidence that mismapping is an issue at either the DNA (assessed in Chen *et al*. 2024) or RNA level. For example, in five randomly chosen samples, >88% of reads map uniquely to *RNU4-2*.

Referee #4

Supplementary Table 1: Please include definitions of abbreviations, especially for information in the headers.

We now define all abbreviations used in Supplementary Table 1 within the table legend.

RNA-sequencing: There is RNA-seq cluster analysis described in the Methods and data shown in Fig 3D (19 ReNU samples and 20 controls or the PCA of the PSI values). Please ensure that the RNAseq data are publicly available and indicate how to access datasets in the Data Availability section.

These data are publicly available in EGA. The following has been added to the Data Availability section to detail this:

> "RNA-sequencing data (**Fig. 3D**) were taken from Nava *et al*. Nature Genetics 2025 and are available in the European Genome–Phenome Archive (EGA, http://www.ebi.ac.uk/ega; study accession EGAS50000000889)."
